# Dietary sugars silence the master regulator of carbohydrate utilization in human gut Bacteroides species

**DOI:** 10.1080/19490976.2023.2221484

**Published:** 2023-06-26

**Authors:** Victoria H. Pearce, Eduardo A. Groisman, Guy E. Townsend

**Affiliations:** a Biochemistry & Molecular Biology, Penn State College of Medicine, Hershey, PA, USA; b Penn State Microbiome Center, Pennsylvania State University, State College, PA, USA; c Center for Molecular Carcinogenesis and Toxicology, Pennsylvania State University, State College, PA, USA; d Microbial Pathogenesis, Yale University School of Medicine, New Haven, CT, USA; e Microbial Sciences Institute, Yale University, New Haven, CT, USA

**Keywords:** Bacteroides, dietary sugar, gut microbiome, carbohydrate utilization, fructose, glucose, transcription regulation

## Abstract

The mammalian gut microbiota is a critical human health determinant with therapeutic potential for remediation of many diseases. The host diet is a key factor governing the gut microbiota composition by altering nutrient availability and supporting the expansion of distinct microbial populations. Diets rich in simple sugars modify the abundance of microbial subsets, enriching for microbiotas that elicit pathogenic outcomes. We previously demonstrated that diets rich in fructose and glucose can reduce the fitness and abundance of a human gut symbiont, *Bacteroides thetaiotaomicron*, by silencing the production of a critical intestinal colonization protein, called Roc, via its mRNA leader through an unknown mechanism. We have now determined that dietary sugars silence Roc by reducing the activity of BT4338, a master regulator of carbohydrate utilization. Here, we demonstrate that BT4338 is required for Roc synthesis, and that BT4338 activity is silenced by glucose or fructose. We show that the consequences of glucose and fructose on orthologous transcription factors are conserved across human intestinal Bacteroides species. This work identifies a molecular pathway by which a common dietary additive alters microbial gene expression in the gut that could be harnessed to modulate targeted microbial populations for future therapeutic interventions.

## Introduction

The gut microbiota compositional changes associated with various human diseases potentiate therapeutic interventions that target distinct microbial populations.^1–8^ Intriguingly, the abundance of individual microbial taxa can be dramatically altered through corresponding changes in the host diet, which directly supplies nutrients to gut microbes.^9–11^ For example, dietary supplementation with arabinoxylan, a hemicellulose, can increase the intestinal abundance of *Bacteroides cellulosilyticus* (*Bc*)^12^ and supplementation with the marine polysaccharide porphyran can increase the abundance of *Bacteroides ovatus* (*Bo*) strains.^13,14^ The advantages exhibited by *Bc* and *Bo* during administration of each respective dietary additive require glycan utilization machinery that enables bacterial consumption of these structurally distinct substrates.^15–17^ Thus, microbial populations that can competitively access the available nutrients are favored and increase in abundance, while populations that cannot are disfavored and consequently decrease in abundance.^13,14^

Dietary components can also influence gut microbial populations by modulating the synthesis of factors necessary for intestinal colonization and host-microbial interactions independently of serving as nutrients.^13,18–20^ For example, the amount of Roc, a gut colonization factor from *Bacteroides thetaiotaomicron* (*Bt*), dramatically decreases upon host consumption of fructose and glucose-rich diets although Roc is dispensable for growth on either substrate.^19^ Roc silencing occurs independently of transcription initiation at the *roc* promoter but requires the 54 nucleotide 5’ leader region of the *roc* mRNA.^19^ Similarly, abundant dietary glucose consumption reduces the amount of BT4295, a *Bt* cell-surface protein that elicits beneficial host immune responses but is dispensable for intestinal colonization.^20^ Roc and BT4295 are each encoded within distinct polysaccharide utilization loci that putatively target unknown host-derived glycans.^21,22^ Thus, human dietary components, such as glucose and fructose control both gut microbiota composition and behavior by altering gene expression in intestinal microbes. Glucose and fructose are highly abundant in the diets of industrialized populations^23^, can reduce the abundance of beneficial microbes, such as *Bt*,^19^ and remodel gut microbial populations into those that elicit pathogenic consequences.^10,24,25^ However, how these sugars exert their effects on gut microbes remains largely unknown.

Carbohydrate utilization genes are regulated by various mechanisms in intestinal Bacteroides species, including an extensive repertoire of glycan-responsive transcription factors,^26–31^ an intricate network of sRNAs,^32–34^ RNA-binding proteins,^35,36^ DNA inversions,^37,38^ and activation of the master regulator of carbohydrate utilization, called BT4338 in *Bt* (originally identified as MalR)^39^, and is conserved in the Bacteroides genus.^26,27^ BT4338 controls the expression of many mono- and polysaccharide utilization genes and several other factors necessary for successful gut colonization.^26,27^ *BT4338*-dependent mRNAs dramatically increase when *Bt* is subjected to carbon limitation for 10 min in laboratory media, but only a fraction of the 464 differentially transcribed genes exhibit BT4338 binding to their putative promoter regions.^27^ This suggests that BT4338 controls target gene transcription both directly and indirectly.

Here, we used Roc protein abundance as a reporter to elucidate how glucose and fructose target regulatory pathways in *Bt* and identified *BT4338* as necessary for controlling Roc amounts following carbon limitation and during growth in substrates other than glucose or fructose. We establish that BT4338 indirectly controls Roc amounts via the *roc* mRNA leader, which is necessary for and sufficient to confer *BT4338*-dependent control to a heterologous gene. Furthermore, we show that BT4338 governs Roc abundance independently of an alternative translation elongation factor, EF-G2^40^, whose mRNA levels are regulated by BT4338 and increase dramatically during carbon limitation.^27^ Finally, we demonstrate that glucose and fructose exert rapid, dramatic, and dominant silencing of BT4338-dependent genes *in vitro* and that these effects are consistent across distinct gut Bacteroides species. Importantly, BT4338 protein levels remain stable, indicating that the metabolism of glucose or fructose alters the production of an unidentified signal that controls BT4338 activity. Our findings collectively indicate that abundant dietary sugar consumption by the host silences BT4338 activity *in vivo*, thereby modulating the production of microbial factors necessary for host interactions and fitness in the mammalian gut.

## Results

### BT4338 is necessary for Roc synthesis

**Figure 1.**
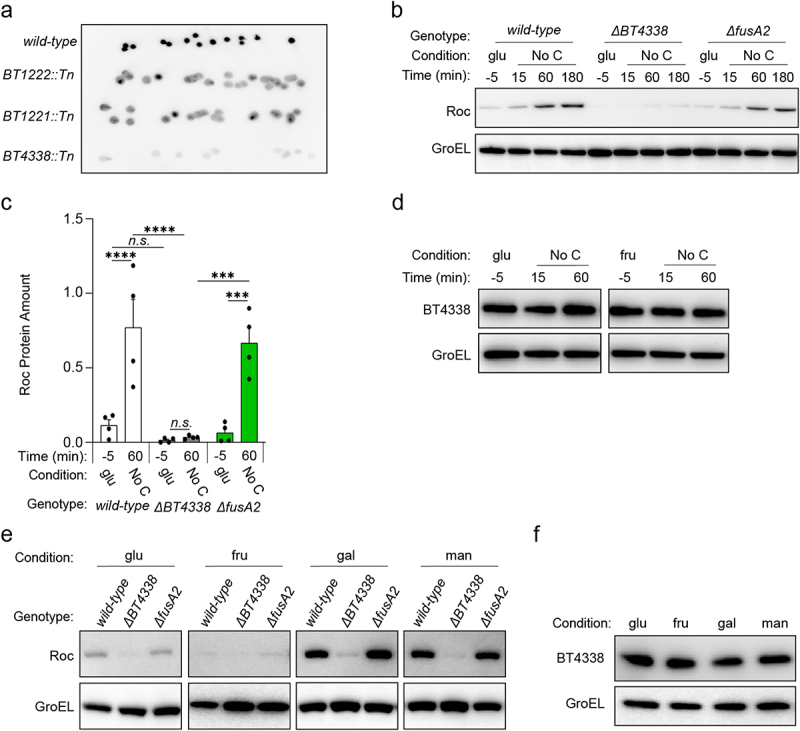
BT4338 is required for Roc synthesis. (a) Colony blot analysis of selected mutants harboring transposon insertions in *BT1222* (GT3148), *BT1221* (GT3150), or *BT4338* (GT3151) compared to a control strain (GT1663) grown on solid minimal media containing rhamnose and galactose. Each strain is represented by at least 10 different isogenic colonies. (b) Western blot analysis of Roc from *wild-type* (GT593) or *Bt* strains deficient for *BT4338* (GT1234) or *fusA2* (GT1310) during growth in glucose (glu; −5) or 15-, 60-, and 180-minutes following exposure to carbon limitation (No C). (c) Quantified western blot analysis of wild-type (GT593; black), or *Bt* strains deficient for *BT4338* (GT1234; gray), or *fusA2* (GT1310; green) during growth in glucose (glu; −5) and 60-min following exposure to carbon limitation (No C) (n = 4 biological samples; error bars represent SEM; P values derived from two-way ANOVA; n.s. indicates *P* values ≥0.05; ***P < 0.001; ****P < 0.0001). (d) Western blot analysis of BT4338 (GT1481) during mid-exponential growth in glucose (glu; −5) or fructose (fru; −5) or 15-, and 60-minutes following exposure to carbon limitation (No C). (e) Western blot analysis of Roc from the strains described in (b) following mid-exponential growth in glucose (glu), fructose (fru), galactose (gal), or mannose (man). (f) Western blot analysis of BT4338 (GT1481) during mid-exponential growth in glucose (glu), fructose (fru), galactose (gal), or mannose (man). Blots were probed using anti-HA and anti-GroEL antibodies.

We sought to determine how *Bt* controls Roc levels by identifying genes required for its synthesis. We screened 8,000 mutants harboring random transposon insertions for reduced Roc levels *in vitro* using colony blotting from strains cultured on solid media containing 0.25% each of rhamnose and galactose. This condition was selected because Roc levels are readily detectable (Figure 1a) and mutations that could disable growth on one monosaccharide were unlikely to prevent growth on the other. We identified seven mutants exhibiting reduced Roc levels and subsequent semi-random PCR revealed that these strains contained an insertion in one of the three different open reading frames (ORF): *BT1222*, *BT1221*, or the master regulator of carbohydrate utilization, *BT4338* (Figure 1a).^27^ On solid media, *BT1222* and *BT1221* mutants exhibited heterogeneous signal intensity within and between colonies, suggesting local differences in Roc abundance, whereas *BT4338* mutants displayed uniform reductions in Roc amounts in all colonies (Figure 1a). We engineered *Bt* strains with in-frame, chromosomal deletions of *BT1222*, *BT1221*, or *BT4338* and measured Roc amounts by western blotting following carbon limiting conditions, which were previously shown to increase Roc levels^19^ and BT4338 binding to target promoters.^27^

Roc amounts increased 5.8-fold and 15.6-fold, respectively, in *wild-type Bt* following a 60-min exposure to carbon limitation conditions following mid-exponential growth in media containing either glucose (Figure 1b,c) or fructose (Fig. S1A and S1B) as the sole carbon source. Compared to *wild-type Bt*, an isogenic *BT4338*-deficient strain exhibited similar Roc levels during growth in glucose (Figure 1b) or fructose (Fig. S1A), which increased 2.5- and 2.3-fold after 60 min in carbon limitation conditions (Figure 1c and Fig. S1B, respectively). Conversely, strains lacking either *BT1222* or *BT1221*, which putatively mediate two discreet steps in the oxidative pentose phosphate pathway (Fig. S2A), exhibited 5.6- and 7.3-fold increased Roc amounts after 60 min that were significantly lower than *wild-type Bt* under identical conditions (Fig. S2B and C). Additionally, a strain lacking *BT1220*, which is putatively required for an intermediate metabolic step between *BT1221* and *BT1222* (Fig. S2A), also exhibited lower Roc amounts than *wild-type Bt* following carbon limitation (Fig. S2C), indicating that the oxidative pentose phosphate pathway is involved in controlling Roc abundance. However, we focused our investigation on understanding how BT4338 controls Roc because it elicits the strongest effect across all conditions.

We determined that BT4338 protein amounts were similar during mid-exponential phase growth in either glucose or fructose, and subsequent exposure to carbon limiting conditions (Figure 1d and Fig. S3A and S3B). These results indicate that carbon limitation increases Roc by stimulating BT4338 activity rather than increasing its protein amount and reciprocally suggests that growth in glucose or fructose reduce BT4338 activity. Consistent with this notion, relative to *wild-type Bt* cells grown in galactose and mannose, Roc amounts were 12.0- and 6.6-fold lower, respectively, in fructose grown cells and 5.8- and 3.2-fold lower, respectively, in glucose grown cells (Figure 1e and Fig. S3C) agreeing with previous results.^19^ By contrast, a *BT4338*-deficient strain exhibited similar Roc amounts across *Bt* cells grown in glucose, fructose, galactose, or mannose (Figure 1e and Fig. S3C), which were 3.8-, 4.0-, 17.2-, and 10.3-fold lower than those from *wild-type Bt* in each respective condition (Fig. S3C). Finally, increased Roc amounts during growth in each monosaccharide are not the result of altered BT4338 protein abundance, which were identical during growth in all four carbon sources (Figure 1f and Fig. S3D). Cumulatively, these data demonstrate that *Bt* controls Roc amounts in response to carbon limitation and carbohydrate metabolism by modulating BT4338 activity.

### BT4338 indirectly controls Roc via its mRNA leader

**Figure 2.**
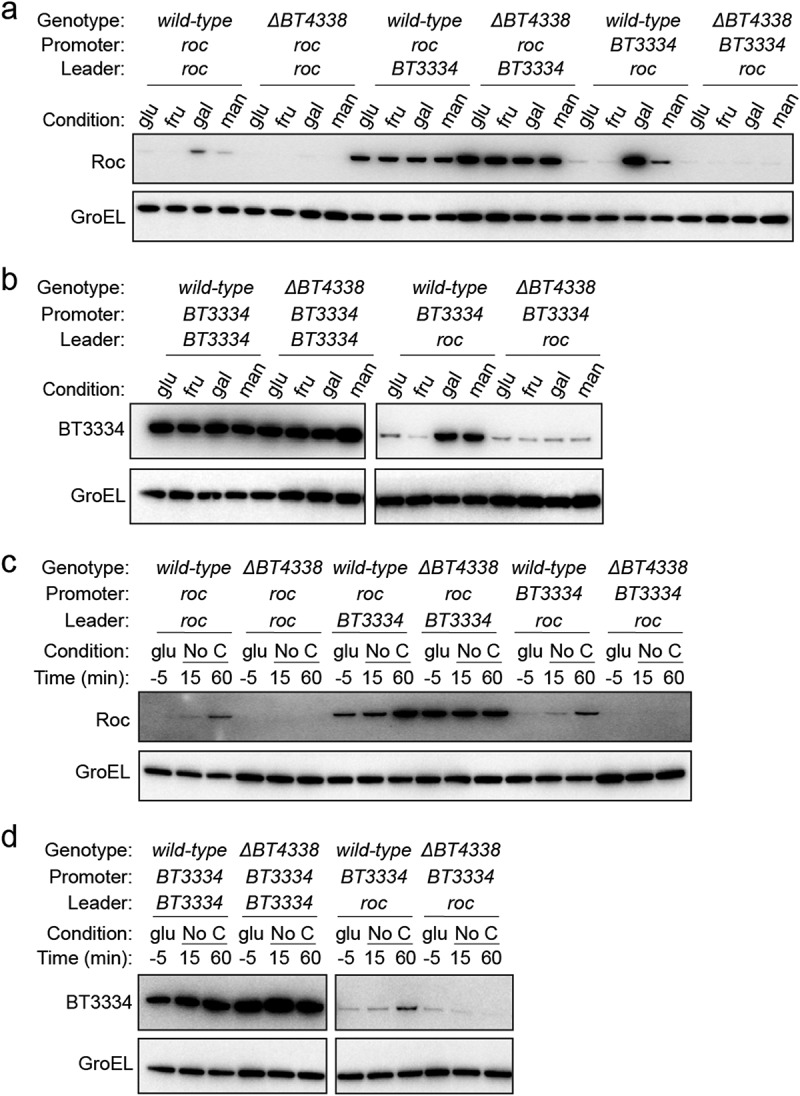
BT4338 governs Roc levels via its 5’ mRNA leader. (a) Western blot analysis of Roc from engineered strains harboring the *roc* leader and ORF positioned downstream of its native (GT530) or heterologous (GT670) promoters, or strains with the native *roc* promoter upstream of a heterologous 5’ leader region (GT665) in strains encoding *BT4338* or a *BT4338*-deficient background (GT3509, GT3511, and GT3510, respectively) grown in minimal media containing 0.5% glucose (glu), fructose (fru), galactose (gal), or mannose (man) as the sole carbon source. (b) Western blot analysis of BT3334 from engineered strains harboring the *BT3334* promoter and ORF flanking either the *BT3334* (GT534) or *roc* (GT663) leaders in isogenic strains encoding *BT4338* or in a *BT4338*-deficient background (GT3512 and GT3514, respectively) grown in minimal media containing 0.5% glucose (glu), fructose (fru), galactose (gal), or mannose (man). (c) Western blot analysis of Roc from strains described in (a) during mid-exponential growth in glucose (glu, −5) or 15-, and 60- minutes following exposure to carbon limitation conditions. (d) Western blot analysis of BT3334 from strains described in (b) during mid-exponential growth in glucose (glu, −5) or 15-, and 60-minutes following exposure to carbon limitation conditions. Blots were probed using anti-HA and anti-GroEL antibodies.

We examined Roc amounts in *BT4338*-deficient strains harboring constructs encoding the *roc* ORF preceded either by its native leader or by the sugar-resistant leader upstream of the heterologous gene, *BT3334*, that enables Roc production even in the presence of fructose or glucose.^19^ As previously demonstrated, the strain that includes the *BT3334* leader displayed similar Roc amounts when grown in either glucose, fructose, galactose, or mannose (Figure 2a and S4A).^19^ This contrasts a strain encoding the *roc* ORF downstream of the native *roc* leader that exhibits 19.4- and 12.1-fold more Roc in galactose and mannose, respectively, compared to fructose grown cells and 3.8- and 2.4-fold more Roc compared to glucose grown cells (Figure 2a and Fig. S4B). These results independently demonstrate that the silencing effect of fructose or glucose on Roc protein amounts requires its mRNA leader. The *BT4338*-deficient strain encoding *roc* downstream of its native promoter and leader displayed similar Roc amounts in glucose or fructose grown cells but 44.6- and 9-fold lower abundances in galactose and mannose, respectively, than those from a *wild-type* strain harboring an identical construct (Figure 2a and Fig. S4A). Furthermore, a strain encoding the *BT3334* promoter preceding the *roc* leader exhibited 34.9- and 11-fold greater Roc amounts in galactose and mannose, respectively, compared to fructose grown cells and 6.4- and 2-fold more Roc, compared to glucose grown cells (Figure 2a and Fig. S4C). In this strain, increased Roc protein abundances in galactose or mannose compared to fructose or glucose grown cells required *BT4338* (Figure 2a and Fig. S4C), demonstrating that the *roc* leader is necessary for *BT4338*-dependent Roc production while the promoter is dispensable.

To determine whether the *roc* leader confers fructose- and glucose-dependent silencing to the heterologous gene, *BT3334*, which is synthesized in the presence of glucose or fructose^19^ independently of BT4338 (Figure 2b and S4D), we used a strain where the *BT3334* ORF was expressed by its native promoter but preceded by the *roc* leader. This strain exhibited BT3334 protein amounts that were 11- and 6.7-fold higher when grown in galactose or mannose, respectively, compared to fructose grown cells and 2.3- and 1.4-fold higher compared to glucose grown cells (Figure 2b and S4E). Increased BT3334 amounts in this strain required *BT4338* (Figure 2b and S4E) demonstrating that the *roc* leader confers *BT4338*-dependent control of the downstream ORF, suggesting that growth in glucose or fructose reduce the corresponding protein abundances by silencing BT4338 activity. Accordingly, when either the *roc* or *BT3334* ORFs were encoded immediately downstream of the *roc* leader, removing glucose from the medium increased the corresponding protein amounts by 20.2-fold (Figure 2c and Fig. S4F) and 3.5-fold, respectively (Figure 2d and Fig. S4G). Increased Roc amounts during carbon limitation required BT4338 when the *roc* ORF was preceded by its native leader regardless of the preceding promoter (Figure 2c and Fig. S4H). While strains encoding the *BT3334* leader upstream of the *roc* ORF exhibited 2.9-fold increased Roc abundance 60 min following exposure to carbon limitation, Roc amounts were increased in a *BT4338*-deficient background during growth in glucose (Figure 2c and Fig. S4I), which resembled BT3334 protein amounts when the *BT3334* ORF was positioned downstream of its native leader (Figure 2d and Fig. S4J). Collectively, these data demonstrate the *roc* mRNA leader is sufficient to confer BT4338-dependent synthesis of the downstream ORF, which is silenced by glucose and fructose independently of the upstream promoter.

**Figure 3.**
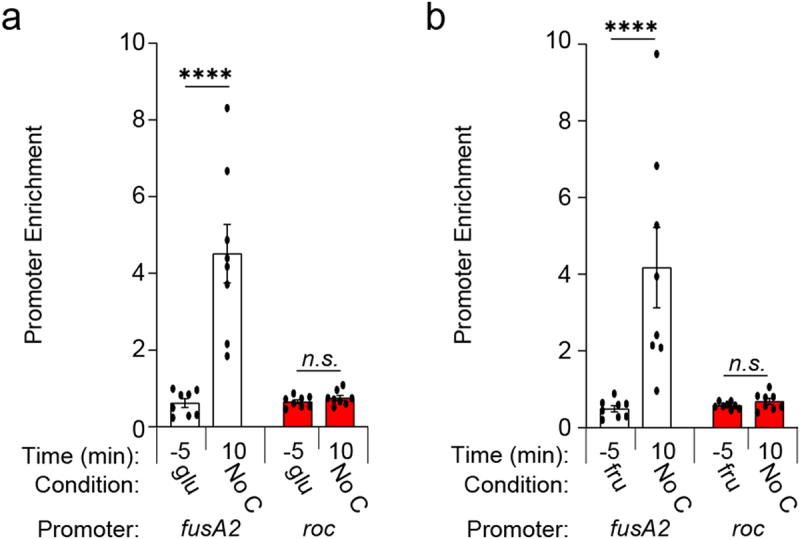
BT4338 DNA binding activity is stimulated during carbon starvation. (a – b) ChIP analysis of the *fusA2* (*BT2167*; black) and *roc* (*BT3172*; red) promoter regions from *wild-type Bt* (GT1481) cells grown to mid-exponential phase in minimal media containing (a) glucose (glu; −5) or (b) fructose (fru; −5) and 10 minutes following exposure to carbon limitation conditions (n = 8 biological samples; error bars represent SEM; *P* values derived from two-way ANOVA; n.s. indicates *P* values ≥0.05; ****P < 0.0001).

Carbon limitation increases Roc abundance (Figures 1b and 2c)^19^ and stimulates BT4338 binding to chromosomal regions throughout the *Bt* genome.^27^ While our previous RNAseq study revealed that *roc* transcript levels were 2.6-fold lower in a *BT4338*-deficient strain 10 minutes following carbon limitation, a corresponding ChIP-seq analysis did not detect BT4338 binding to regions upstream of the *roc* ORF under the same conditions.^27^ This contrasts targets like *fusA2*, whose promoter occupancy dramatically increased 10 min after exposure to carbon limiting conditions, resulting in a *BT4338*-dependent 238-fold increase in the corresponding mRNA levels.^27^ In agreement with these results, we determined that enrichment of the *fusA2* promoter increased 7.3- and 8.4-fold by 10 min following carbon limitation compared to immunoprecipitation of BT4338 from glucose (Figure 3a) or fructose (Figure 3b) grown *Bt*. Although BT4338 levels remain constant throughout carbon limitation following growth in glucose or fructose (Figure 1d), *roc* promoter enrichment was not detected under these conditions (Figure 3a,b) and the *roc* promoter lacks sequences resembling the BT4338 consensus.^41^ Thus, BT4338 controls Roc amounts by a mechanism other than directing *roc* transcription initiation.

### EF-G2 and other BT4338 regulated products are dispensable for Roc synthesis

We hypothesized that BT4338 controls Roc amounts by regulating transcription of an unknown factor(s) involved in its synthesis. The most highly induced *BT4338*-dependent gene during carbon limitation conditions is *fusA2* (*BT2167*), which encodes a non-essential, alternative translation elongation factor G (EF-G2) that enables GTP-independent translation^40^ and is critical for mammalian intestinal colonization.^27^ Because BT4338 is required for synthesis of both EF-G2 and Roc, and BT4338-dependent control of Roc protein is indirect and mediated via the *roc* 5’ mRNA leader, we reasoned that BT4338-dependent EF-G2 expression may control Roc amounts by governing its translation in a leader-dependent manner. However, a *fusA2*-deficient strain exhibited 10.3- and 9.3-fold increased Roc amounts following a 60-min exposure to carbon limitation from glucose or fructose, respectively, which resembled the 7.2- and 11.7-fold increases exhibited by *wild-type Bt* (Figure 1b,c and Fig. S1A and S1B). Furthermore, a *fusA2*-deficient strain displayed similar Roc amounts as *wild-type Bt* in all growth conditions with 15.5- and 10.5-fold increases over fructose in either galactose or mannose, respectively (Figure 1e and Fig. S3C). Cumulatively, these data demonstrate that EF-G2 is dispensable for Roc synthesis.

To explore the role of additional BT4338 regulated genes in controlling Roc abundance, we examined the consequences of inactivating the *BT4338*-dependent polysaccharide utilization locus (PUL), *BT4299-BT4295*, and putative methylmalonyl-CoA biosynthetic genes, *BT1450-BT1448*. However, both mutants displayed similarly increased Roc amounts compared to *wild-type Bt* after carbon limitation for 60 min (Fig. S5A-C). We also examined Roc levels in strains lacking *BT2131*, which encodes a conserved hypothetical protein, that could putatively silence Roc levels in the absence of *BT4338* because the *BT2131* transcript increases 57.3-fold in a *BT4338*-deficient strain during growth in glucose.^27^ However, Roc amounts were indistinguishable between a *BT4338* mutant and a strain lacking both *BT4338* and *BT2131* (Fig. S5A and S5D), and between *wild-type Bt* and a strain lacking *BT2131* alone (Fig. S5A and S5D). Thus, BT4338 controls Roc abundance by an unidentified gene product(s).

### Candidate Bt sRNAs are insufficient to control Roc levels

*Bt* produces hundreds of small RNAs (sRNA) that have established roles in regulating gene products involved in carbohydrate utilization.^32–34^ Furthermore, a subset of *Bt* sRNAs increase in abundance following exposure to carbon limitation *in vitro*^34^ and are positioned proximally to BT4338 binding sites.^27^ Computational analysis revealed that three of these sRNAs, *BTnc140*, *BTnc195*, and *BTnc364* exhibit complementarity to the *roc* leader (Fig. S6A), suggesting a role in controlling Roc amounts. However, *Bt* strains engineered to over-produce either *BTnc140*, *BTnc195*, or *BTnc364* (Fig. S6B) exhibited indistinguishable Roc amounts compared to those of a control strain during mid-exponential phase growth in glucose (Fig. S6C and S6D). These data indicate that increased expression of three candidate sRNAs cannot increase Roc protein amounts.

### Glucose and fructose rapidly and dominantly silence BT4338 activity

**Figure 4.**
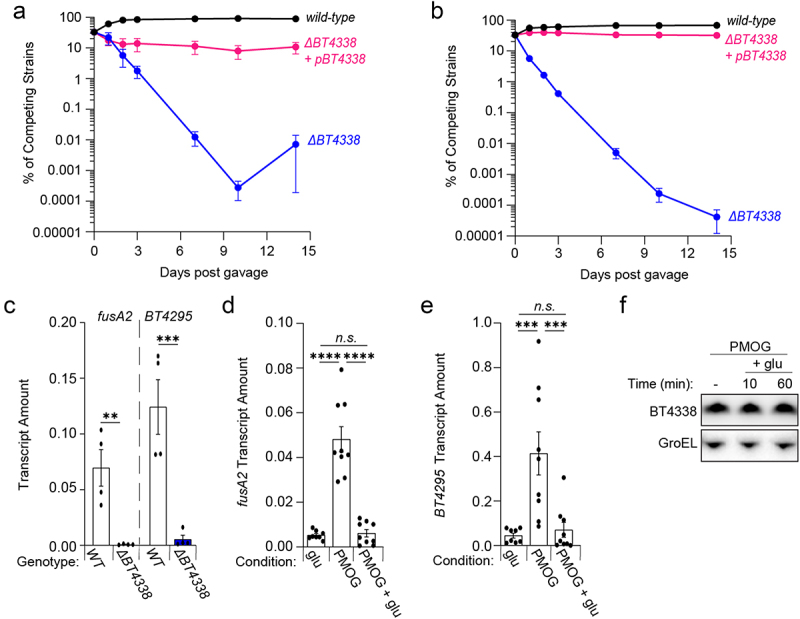
Dietary sugars silence BT4338 activity. (a & b) the relative abundances of bar-coded strains *wild-type* (GT3361; black) or *BT4338*-deficient *Bt* strains harboring an empty vector (GT3522; blue) or complementation plasmid (GT3363; pink) at the indicated times following gavage with equal cfus of each strain into germ-free mice fed (a) a sugar-rich diet or (b) a standard diet (n = 5; error bars represent SEM). (c) qPCR analysis of *fusA2* (*BT2167*) or *BT4295* transcript levels measured in *wild-type* (GT593; black) or *BT4338*-deficient (GT1234; gray) *Bt* strains during mid-exponential growth in 1% PMOG (n = 4 biological samples; error bars represent SEM, *P* values derived from two-way ANOVA; n.s. indicates *P* values ≥ 0.05; **P < 0.01 ***P < 0.001). (d – e). qPCR analysis of (d) *fusA2* (*BT2167*) or (e) *BT4295* transcript levels measured in *wild-type Bt* (GT23) during mid-exponential growth in either 0.5% glucose (glu) or 1% PMOG, and 10-minutes following the addition of 0.2% glucose to the PMOG grown cells. (n = 9 biological samples; error bars represent SEM, *P* values were calculated using two-way ANOVA; n.s. indicates *P* values ≥0.05; ***P < 0.001; ****P < 0.0001). (f) Western blot analysis of BT4338 from cells (GT1481) grown in 1% PMOG or 10 and 60-minutes following the addition of glucose to 0.2%. Blot was probed using anti-HA and anti-GroEL antibodies.

Host consumption of abundant dietary glucose and fructose reduce the levels of two *BT4338*-dependent products, Roc^19^ and BT4295 *in vivo*.^20^ Because *BT4338* is a critical determinant of mammalian intestinal colonization,^27^ we hypothesized that host consumption of sugar-rich chow would silence BT4338 activity in *wild-type Bt*, thereby reducing the competitive defect exhibited by a *BT4338*-deficient strain. An independently constructed *Bt* strain lacking *BT4338* was 1.3 × 10^4^-fold lower in abundance than *wild-type Bt* 10 days following introduction into germ-free mice fed a sugar-rich diet (Figure 4a). The introduction of a plasmid-borne copy of BT4338 complemented this mutant, which exhibited 1.5 × 10^3^-fold greater abundance than the *BT4338*-deficient strain (Figure 4a). The *BT4338*-deficient strain exhibited a 1.6 × 10^6^-fold lower abundance than the *wild-type Bt* strain in mice fed a standard, low sugar, high plant polysaccharide diet (Figure 4b), indicating that *BT4338* is required for intestinal colonization regardless of host dietary sugar consumption. These results agree with previous reports demonstrating that *Bt* mutants harboring insertions in *BT4338* exhibit severe competitive defects for murine gut colonization in hosts fed either standard low in sugar or glucose and fructose-rich chows^12,27^. Thus, this transcription factor performs critical regulatory roles in the mammalian gut independently of dietary composition.^27^

To determine whether fructose or glucose exert dominant silencing effects on BT4338 activity, thereby reducing target gene transcription, we compared *fusA2* transcript abundances during growth on glucose or a glycan mixture derived from the porcine gastric mucosa (PMOGs). PMOGs support the growth of *wild-type* and *BT4338*-deficient *Bt* strains (Fig. S7A)^16^ and elicit 9.3- and 9.1-fold increases in *fusA2* and *BT4295* transcripts, respectively, in a *BT4338*-dependent manner (Figure 4c). Glucose dominantly silences BT4338 activity because *fusA2* and *BT4295* transcripts decreased by 7.9- and 5.9-fold, respectively, 10 min following the introduction of 0.2% glucose to *Bt* cells grown to mid-exponential phase in 1% PMOGs (Figure 4d,e, respectively). Likewise, the addition of fructose to PMOG-grown *Bt* cells also decreased *fusA2 and BT4295* transcripts by 5.4- and 14.8-fold, respectively, after 60 min although no change was detected by 10 min (Fig. S7B and S7C, respectively), likely because additional time is necessary for optimal synthesis of gene products necessary for fructose consumption.^17,42^ Importantly, BT4338 protein levels remained constant after 60 min following glucose addition, indicating that transcription factor activity is silenced (Figure 4f and Fig. S7D). Collectively, these data indicate that available glucose and fructose can rapidly modulate BT4338 activity even in the presence of other growth substrates.

### BT4338 orthologs govern a partially conserved regulon

**Figure 5.**
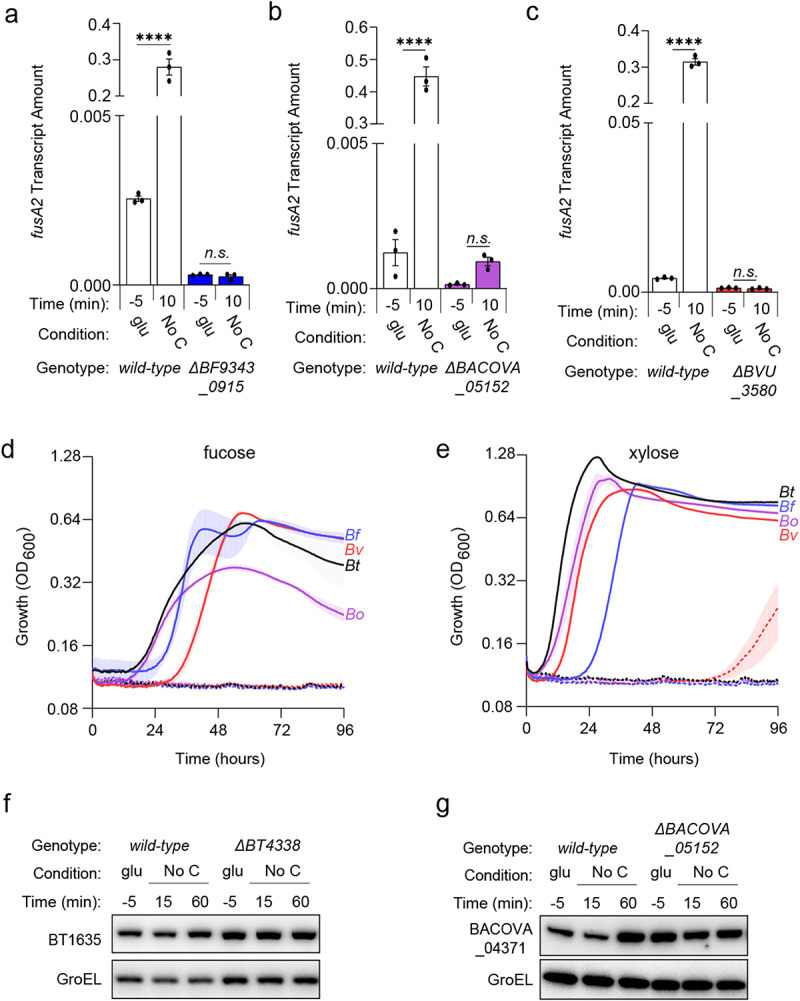
BT4338-orthologs govern a conserved regulon among prominent Bacteroides species. (a) the transcript level of *B.* *fragilis fusA2* (*BF9343_3536*) was measured in *wild-type* (ATCC 25285; black) or a *BF9343_0915*-deficient (GT2520; blue) *B.* *fragilis* strain grown in glucose (glu; −5) and 10-minutes after exposure to carbon limitation (No C) (n = 3 biological samples; error bars represent SEM, P values were calculated using two-way ANOVA; n.s. indicates *P* values ≥0.05; ****P < 0.0001). (b) the transcript level of *B.* *ovatus fusA2* (*BACOVA_03178*) was measured in *wild-type* (*ATCC 8483*; black) or *BACOVA_05152*-deficient (GT2413; purple) strains grown in glucose (glu; −5) or 10-minutes after exposure to carbon limitation (No C) (n = 3 biological samples; error bars represent SEM, *P* values derived from two-way ANOVA; n.s. indicates *P* values ≥ 0.05; ****P < 0.0001). (c) the transcript level of *B.* *vulgatus fusA2* (*BVU_0017*) was measured in *wild-type* (*ATCC 8482*; black) or *BVU_3580*-deficient (GT2399; red) strains grown in glucose (glu; −5) or 10-minutes after exposure to carbon limitation (No C) (n = 3 biological samples; error bars represent SEM, *P* values derived from two-way ANOVA; n.s. indicates *P* values ≥ 0.05; ****P < 0.0001). (d – e). Growth of bar-coded *wild-type* (solid lines) *B.* *thetaiotaomicron* (*Bt*; GT3361; black), *B.* *fragilis* (*Bf*; GT3551; blue), *B.* *vulgatus* (*Bv*; GT3367; red), or *B.* *ovatus* (*Bo*; GT3364; purple) or bar-coded isogenic BT4338-ortholog-deficient strains (dashed lines; GT3522, GT3555, GT3643, GT3553, respectively) in minimal media containing 0.5% (d) fucose or (e) xylose (n = 4 biological samples; error bars represent SEM). (f) Western blot analysis of BT1635 amounts from *wild-type* (GT4372) or a *Bt* strain deficient for *BT4338* (GT4373) during growth in glucose (glu; −5) or 15-, or 60-minutes following exposure to carbon limitation (No C). (g) Western blot analysis of BACOVA_04371 from *wild-type Bo* (GT4362) or a strain deficient for *BACOVA_05152* (GT4369) during growth in glucose (glu; −5) or 15-, or 60-minutes following exposure to carbon limitation (No C). Blots were probed using anti-HA and anti-GroEL antibodies.

BT4338 is conserved among numerous Bacteroides species^12^ including *B. fragilis* (*Bf, BF9343_0915*), *B. ovatus* (*BACOVA_05152*), and *B. vulgatus* (*Bv, BVU_3580*), which share 84.1, 96.1, and 77.3% amino acid sequence identity with BT4338, respectively. Exposing each species to carbon limitation for 10 minutes elicited 109.9-, 360.9-, and 75.6-fold increased transcription of their corresponding *fusA2* orthologs, *BF9343_3536, BACOVA_03178*, and *BVU_0017* (Figure 5a-c, respectively), whose products share 89.1, 97.9, and 86.9% amino acid sequence identity to *BT2167*, respectively. *Bf, Bo*, and *Bv* strains deficient for their respective BT4338-orthologs are unable to increase *fusA2* transcription in response to carbon limitation conditions (Figure 5a-c, respectively).^27^ Furthermore, *Bf, Bo*, and *Bv* mutants deficient for this transcription factor are unable to grow on fucose or xylose (Figure 5d,e, respectively) but can grow on glucose or fructose (Fig. S8A and S8B, respectively). Together, these data demonstrate a conserved regulon including control of *fusA2* transcription and distinct carbon utilization genes across Bacteroides species.

To determine whether BT4338-orthologs control the abundances of Roc homologs, we examined the amounts of BT1635, which is a putative hybrid two-component system (HTCS) exhibiting 74.1% amino acid sequence identity to Roc. Like Roc, BT1635 regulates the expression of linked PUL genes in response to unknown glycans.^22,43^ In contrast to Roc, BT1635 protein amounts are similar during growth in 8 different substrates, including glucose or fructose.^19^ Both *wild-type* and *BT4338*-deficient strains display indistinguishable BT1635 abundances either during mid-exponential phase growth in glucose or following carbon limitation for 60 min (Figure 5f and S9A), indicating that BT4338 does not control this protein even though it shares high sequence identity to Roc. We also examined the abundance of BACOVA_04371, a HTCS in *Bo* that shares 73.3% identity to Roc and 80.6% identity to BT1635. In contrast to both BT1635 and Roc, BACOVA_04371 is not predicted to control PUL gene expression^22,43^ and its abundance increased 3-fold in a *BACOVA_05152*-deficient strain compared to *wild-type Bo* grown in glucose (Figure 5g and S9B). BACOVA_04371 levels remained constant following exposure to carbon limitation for 60 min, which was similar in *wild-type Bo* and the *BACOVA_05152*-deficient strain (Figure 5g and S9B). This indicates that control of Roc-like protein amounts by BT4338-orthologs is unpredictable based on sequence identity across *Bt* and *Bo*, and the regulatory outcomes can differ between species.

### Dietary sugar silences BT4338-ortholog activity across prominent bacteroides species

**Figure 6.**
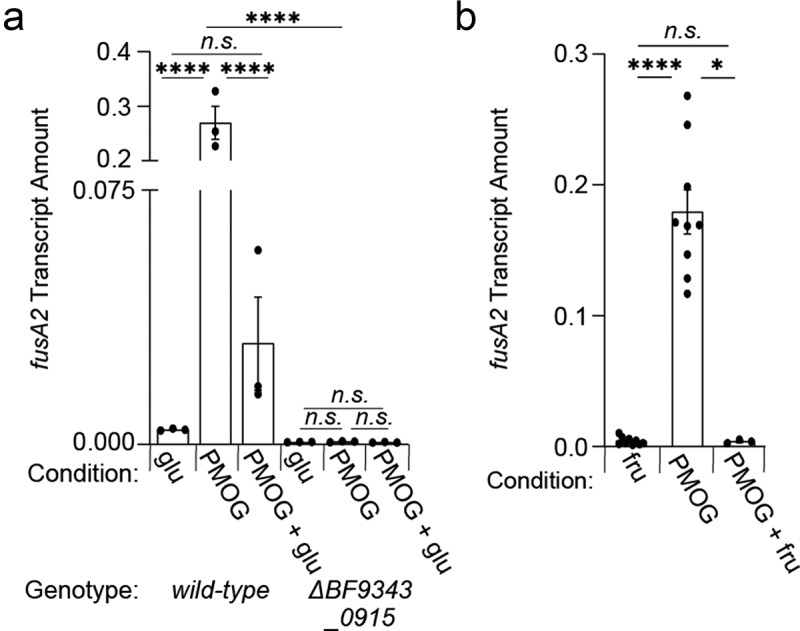
A BT4338-ortholog is silenced by dietary sugar addition in *B.* *fragilis*. (a) *Bf fusA2* (*BF9343_3536*) transcript levels were measured in *wild-type* (ATCC 25285; black) or a *BF9343_0915*-deficient (GT2520; blue) *B.* *fragilis* strain grown in 0.5% glucose, 1% PMOG, or 1% PMOG 10-minutes after addition of 0.2% glucose (n = 3 biological samples; error bars represent SEM, P-values derived from two-way ANOVA; n.s. indicates *P* values ≥ 0.05; ****P < 0.0001). (b) the transcript level of *B. fragilis fusA2* (*BF9343_3536*) was measured in *wild-type* (ATCC 25285) or a *BF9343_0915*-deficient (GT2520) strain during mid-exponential growth in either 0.5% fructose (fru) or 1% PMOG, and 60-minutes following the addition of fructose to 0.2% (n = 9 biological samples for fru and PMOG; n = 3 for PMOG + fru; error bars represent SEM, *P* values derived from one way ANOVA; n.s. indicates *P* values ≥ 0.05; *P < 0.05 ****P < 0.0001).

To determine if glucose and fructose dominantly silence the activity of BT4338 orthologs, we examined *fusA2* transcript levels in *Bf* during mid-exponential phase growth in either glucose or PMOGs, which both support growth of *wild-type* and *BF9343_0915*-deficient strains (Fig. S10). We determined that *fusA2* transcript levels in *wild-type Bf* are 42.2- and 44.9-fold lower during growth in glucose (Figure 6a) or fructose (Figure 6b), respectively, compared to PMOGs as a sole carbon source. Additionally, the *BF9343_0915*-deficient strain exhibited indistinguishable *fusA2* levels during growth in either glucose or PMOGs, which were 6.4- and 335-fold lower than *wild-type Bf* in each respective condition (Figure 6a). The addition of 0.2% glucose to *Bf* cells growing on 1% PMOGs as a sole carbon source reduced *fusA2* transcript levels 24.5-fold after 10 min (Figure 6a), and 44.9-fold after 60 min following fructose addition (Figure 6b). Together, these results demonstrate that the introduction of glucose and fructose rapidly silence BT4338 ortholog activity and reduce target gene transcription in abundant intestinal Bacteroides species.

## Discussion

We have established that a widely distributed transcription factor in human gut bacteria governs carbohydrate utilization (Figure 5d,e) and transcription of the alternative translation elongation factor, EF-G2 (Figure 4c). Moreover, introduction of the abundant human dietary sugars, glucose and fructose, reduce the levels of *BT4338*-dependent transcripts and proteins including *Bt* gene products, Roc (Figure 1b), *fusA2* (Figure 4d and Fig. S7B) and *BT4295* (Figure 4e and Fig. S7C), that mediate host-microbial interactions and are silenced *in vivo* by host consumption of dietary sugars.^19,20^ Furthermore, the addition of glucose or fructose rapidly and dominantly exert these effects on cultured cells consuming host-derived glycans (Figure 4d,e and Fig. S7B and S7C), and conversely, removing fructose or glucose from the growth media dramatically increase target promoter binding (Figure 3a,b) and gene transcription^27^ without altering transcription factor levels (Figure 1d and Fig. S3A and S3B). Collectively, these data indicate that glucose and fructose modulate the activity of this transcription factor, which we propose to rename Cur (Carbohydrate utilization regulator), distinguishing this protein from analogous regulators in Proteobacteria and Firmicutes.^44^ This work identifies a conserved mechanism governing gene expression in dominant human gut bacteria in response to abundant dietary additives, and highlights this pathway as a potential mediator of intestinal disease observed in animals fed a sugar-rich diet.^10^

Cur is an important component of carbon catabolite repression (CCR) in the *Bacteroidetes* because Cur binds to *Bt* carbon utilization gene promoters,^27^ is required for controlling transcription of downstream genes,^27^ and growth on distinct monosaccharides across four representative Bacteroides species (Figure 5d,e).^26^ In other gut bacterial phyla, CCR is mediated by similar transcription factors, such as Crp (also called CAP) in the Enterobacteriaceae and CcpA in Firmicutes, which recognize intracellular signaling molecules that modulate their target promoter-binding activities and direct transcription of genes required for “less-preferred” substrates.^45^ For example, Crp binds cyclic-adenosine monophosphate (cAMP) and CcpA binds a phosphorylated form of HPr, which are differentially synthesized in response to metabolic cues resulting in increased catabolic gene expression.^45^ The activation of Crp and CcpA require components of the phosphoenolpyruvate: sugar transferase system (PTS), which couple monosaccharide transport and phosphorylation to serve as an intracellular indicator of preferred substate availability.^46^ Thus, the transport of preferred growth substrates reduces transcription of genes mediating utilization of “less-preferred” substrates present in the environment by decreasing the activation of Crp and CcpA.^45^

**Figure 7.**
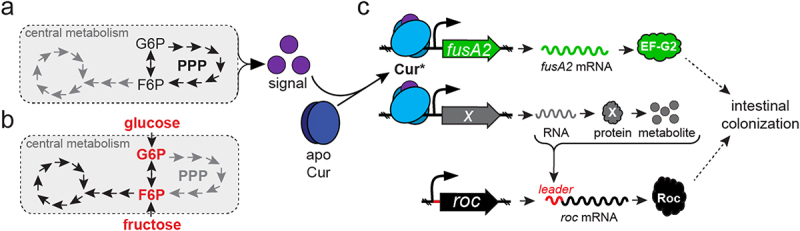
Model depicting the consequences of glucose and fructose on Cur activity. (a) an unknown signal(s) (purple) is putatively synthesized in response to metabolic cues derived from the PPP pathway that converts apo-Cur (dark blue) to Cur* (light blue). (b) Introduction of fructose or glucose modulate central metabolism to reduce Cur signal(s), thereby hindering products necessary for intestinal colonization. (c) Cur* increases transcription of *fusA2* (green) and an unidentified gene (gray), whose product(s) govern Roc abundance by interacting with the *roc* mRNA leader region (red).

Bacteroides species impose CCR by a distinct mechanism(s) because: 1.) all sequenced Bacteroides species lack HPr, CcpA, and PTS orthologs, and 2.) although Bacteroides Cur is classified as a Crp/Fnr-like regulator, these transcription factors exhibit only 18% amino acid sequence identity.^27^ Furthermore, neither cAMP nor its biosynthetic enzyme, adenylate cyclase, have been detected in the Bacteroidetes^47,48^ indicating that Cur activity is controlled by unique signal(s) in this phylum. We hypothesize that perturbations in pentose phosphate pathway (PPP) intermediates modulate this signal (Figure 7a) because *cur* is required for growth on pentose sugars (Figure 5e),^26^ Roc levels increase during growth on pentose sugars,^19,26^ and many PPP genes are dispensable for *in vitro* growth but critical for intestinal colonization, which is similar to the *cur* gene.^12,49,50^ Additionally, two independent genetic screens identified oxidative PPP genes as necessary for synthesis of Cur-dependent products (Figure 1a),^20^ although a third screen identified PPP genes as regulators of Cur-independent gene expression.^51^ We hypothesize that dietary sugars exert CCR by modulating cellular carbon metabolism, thereby reducing the production of the putative Cur signal (Figure 7b), which consequently lowers the synthesis of factors mediating host interactions, including those that function independently of carbohydrate utilization (Figure 7c). Thus, identifying the signal(s) that governs Cur activity in Bacteroides species is imperative to understand how CCR is imposed *in vivo* and to identify strategies to exploit this regulatory system for manipulation of intra-intestinal microbial abundance and metabolism.

Intestinally isolated Bacteroides species can harvest monosaccharides from various complex polysaccharides using expansive repertoires of coordinately regulated glycan detection, importation, and degradation enzymes encoded within PULs.^16,30,42,52–54^ PUL gene transcripts can also be silenced by glucose or fructose and exhibit prioritized expression by a myriad of mechanisms.^26,55^ Here, we demonstrate that in *Bt*, Cur indirectly controls the amount of the PUL-encoded sensor protein, Roc (Figure 2a), and that glucose and fructose silencing of Roc is mediated by a corresponding reduction in Cur activity (Figure 1e,f and Figure 3a). Additionally, Cur is required for increased transcript levels of another PUL-encoded gene, *BT4295*, following exposure to carbon limitation conditions even though Cur does not bind to the putative promoter regions upstream of either *roc* (Figure 3a,b) or *BT4299*, the gene initially transcribed in the PUL containing *BT4295*.^27^ Thus, Cur likely regulates many more genes than previously estimated, including those facilitating polysaccharide utilization, permitting dynamic carbon source prioritization as conditions change within the host.^27,41^ We predict that Cur controls Roc synthesis indirectly by governing transcription of an unknown target gene(s) that interacts with the *roc* leader independently of transcription initiation (Figure 7c). In the *Bacteroidetes* phylum, mRNA leaders can control downstream gene product synthesis by functioning as riboswitches,^56–58^ sRNA targets,^34,59–61^ and interacting with RNA binding proteins.^35,36^ Although the *roc* mRNA leader could function as a riboswitch,^62^ we hypothesize that Cur is more likely to regulate an unknown RNA-binding protein or sRNA that controls Roc production via its leader. Future investigations are necessary to identify species-specific Cur regulons and determine how the abundance of distinct intestinal Bacteroides species are differentially altered in hosts fed glucose and fructose-rich diets.^10^

## Materials and methods

### Bacterial culture conditions

Bacterial strains and plasmids used in this study are listed in Table S1. *Escherichia Coli* strains were cultured on Luria-Bertani (BD). *Bt* strains were cultured on solid brain heart infusion agar (Sigma) containing 5% defibrinated horse blood (Hardy), tryptone-yeast extract-glucose (TYG), or Bacteroides minimal medium, plus individual carbon sources (0.5% weight/volume unless otherwise noted) as previously described (24). All bacterial strains included the following antibiotics where appropriate: 100 μg/mL ampicillin (Sigma), 200 μg/mL gentamicin (Sigma), 2 μg/mL tetracycline (Sigma), or 25 μg/mL erythromycin (Sigma).

### Construction of strains

pNBU2 plasmids were introduced by di-parental mating and att−1 site integration was verified by PCR as previously described.^19^ Strains harboring chromosomal deletions of *BT4338, BACOVA_05152*, or *BVU_3580* were constructed by allelic exchange as previously described.^63^ A strain lacking *BF9343_0915* was constructed by allelic exchange as previously described.^64^ Strains harboring chromosomal deletions of *BT1450-BT1448* or *BT2131* were constructed by allelic exchange as previously described.^65^ Epitope tagging of *BACOVA_04371* and inactivation of *BT4299* genes was performed using the pKNOCK-tetQ vector as described.^54^ Overexpression of candidate sRNAs was achieved using the multicopy plasmid pLYL01 as described.^22^ Primers used in this study are listed in Table S2.

### Generation of Bt transposon insertion library

*E. coli* donor S17–1 strain harboring pSAM-*Bt* (GT671) and recipient *Bt* strain containing a multi-copy plasmid encoding epitope-tagged Roc (GT1663) were cultured overnight to stationary phase in LB and TYG, respectively, containing the appropriate antibiotics.^50^ The *E. coli* strain was diluted 2000-fold into LB media containing ampicillin and the recipient *Bt* strain was diluted 250-fold into 10 ml pre-reduced TYG containing tetracycline. Upon reaching early exponential phase (OD_600_ ~0.3), 1 mL of donor was combined with 10 mL recipient, centrifuged at 7,200 × g for 2 min, and washed with 10 mL of PBS. The pellet was resuspended in the residual volume, spread onto solid BHI-B, incubated aerobically for 3 h, and then anaerobically overnight. 1.0 mL 1× PBS was added and the confluent growth was dislodged from the plate using a cell spreader. The suspension was increased to with 1× PBS to a final volume of 5.0 mL, homogenized by vortex, and 1.0 mL of the resulting cell suspension was spread onto five 245 mm square BioAssay dishes (Nunc) containing solid BHI-B containing 0.2% galactose and the appropriate antibiotics before 48 h of anaerobic incubation. The resulting colonies were collected using a plastic spreader and 12 mL 1× PBS containing 20% glycerol. All re-suspended cells were homogenized by vortex and stored at −80°C as 0.25 mL aliquots prior to colony blotting.

### Colony blotting

One aliquot of the *Bt* library described above was thawed, diluted, and spread onto 150 mm petri dishes containing solid minimal media containing 0.25% galactose and 0.25% rhamnose such that each plate contained approximately 1,000 colonies. Plates were incubated anaerobically for 48 to 72 huntil colonies were readily visible by eye, transferred to nitrocellulose membranes, and immunoblotted as previously described.^19^ Selected colonies were isolated on solid BHI-B prior to cryo-preservation.

### Western blotting

Cell pellets were re-suspended in 375 μl 1× Tris-buffered saline (TBS) containing 1 mM EDTA and 0.5 mg/ml chicken egg lysozyme (Sigma). Cell suspensions were transferred to a 2.0 mL tube containing 0.1 mm Zirconia/Silica beads (BioSpec) and subject to disruption using a Mini-Beadbeater (BioSpec, Bartlesville, OK, USA) at 2,800 rpm for 5 cycles of 40 s with 5-min incubations at 4°C between each cycle. Samples were centrifuged for 2 min at 15,294 × g at 4°C to remove cell debris and the supernatant was reserved. Protein concentration was estimated by measuring absorbance at 280 nanometers using a NanoQuant plate in a Spark plate reader (Tecan, Männedorf, Switzerland). A volume corresponding to 30 μg of protein from each sample was combined with 3 μl 4× LDS buffer (ThermoFisher) containing 100 mM dithiothreitol and subject to heating at 95°C for 5 min. A modified protocol was used for PMOG grown cells to reduce reagent consumption. For this, equivalent 0.75 ODs were calculated from mid-exponential grown cells, pelleted, decanted, and flash frozen. Cell pellets were resuspended in 75 μl lysis buffer containing 50 mM Tris, 1% SDS, and 2× protease inhibitor (P8849, Sigma) before being boiled at 95°C for 5 min. After cooling samples on ice, 25 μl 4× LDS buffer (ThermoFisher) containing 100 mM dithiothreitol was added and samples were incubated at 75°C for 10 min. 15 μl of each sample were loaded onto a 4–12% Bis-tris NuPAGE gel (ThermoFisher) and fractionated for 60 min at 180 V in 1× MOPS running buffer (ThermoFisher) before transferring to a nitrocellulose membrane using an iBlot2 (Invitrogen, Waltham, Massachusetts, USA). The resulting membrane was cut below the 100 kD marker and both portions blocked for 1 hour in 1× TBS with 3% skim milk (BD). The top portion of the membrane was incubated with a 1 to 5,000 dilution of a rabbit anti-HA antibody (Sigma) followed by a 1 to 5,000 dilution of an HRP-conjugated anti-rabbit antibody (GE). GroEL was detected on the bottom portion of the membrane using a 1 to 5,000 dilution of a rabbit anti-GroEL antibody (Sigma) followed by a 1 to 5,000 dilution of an HRP-conjugated anti-rabbit antibody (GE). Membranes were washed before and after addition of secondary antibody with TBS containing 0.05% Tween−20 (Sigma) and rinsed with 1× TBS prior to detection with ECL Prime Western Blotting Detection Reagent Substrate (Cytiva). Blots were quantified using Image Studio Lite (LI-COR Biosciences, Lincoln, Nebraska, USA).

### Quantitative PCR (qPCR)

mRNA was prepared from 1.0 mL of *Bt* cell culture pre-treated with RNA protect (Qiagen) using the RNeasy kit (Qiagen) according to the manufacturer’s instructions. Contaminating DNA was removed using on-column DNase treatment (Qiagen) during purification according to the manufacturer’s instructions. cDNA was synthesized from 1.0 μg of RNA using Superscript VILO IV master mix (ThermoFisher) according to the manufacturer’s instructions. mRNA levels were measured by quantification of cDNA using Fast SYBR Green PCR Master Mix (Applied Biosystems) and primers listed in Table S2 using a QuantStudio 12K Flex Real-Time PCR System (Applied Biosystems, Waltham, Massachusetts, USA). Data were normalized to 16s ribosomal RNA from 1,000-fold diluted cDNA as previously described^26^. qPCR primers are listed in Table S2.

### Monitoring growth of bacterial strains in vitro

Bacteroides strains were growth in TYG medium anaerobically overnight before being diluted 1 to 200 into 100ul of Bacteroides minimal medium containing 0.5% of the carbon sources of interest. For PMOG growth curves, 1.0% PMOG was used. Growth was monitored for 96 h following dilution using an Infinite M Nano plate reader (Tecan, Männedorf, Switzerland) maintained anaerobically. Absorbance at OD600 was measured every 15 min after 5 s of orbital shaking.

### Chromatin immunoprecipitation

ChIP was carried out as previously described.^27^ The abundance of *rpoD, fusA2*, and *roc* promoters were measured in 50-fold-diluted input DNA and 2-fold-diluted IP or control samples by qPCR using primers listed in Table S2. The fold enrichment was calculated as previously described.^66^

### Carbon limitation experiments

*Bt* or *Bo* strains were grown in TYG medium anaerobically overnight before being diluted 1 to 400 into 2.0 mL of Bacteroides minimal medium containing 0.5% glucose. After reaching the stationary phase, the resulting culture was diluted 1 to 50 into pre-reduced medium containing 0.5% glucose or 0.5% fructose and grown to mid-exponential phase (OD_600_ = 0.45 to 0.6), at which time an aliquot was collected by centrifugation, decanted, and immediately placed on dry ice representing the “−5” time points in carbon limitation experiments. The remaining culture was centrifuged at 7,200 × g at room temperature for 3 min in sealed tubes and reintroduced into the anaerobic chamber where the tubes were unsealed, and the supernatant was decanted. Cell pellets were resuspended in an equivalent volume of pre-warmed, pre-reduced minimal medium lacking a carbon source and incubated at 37°C anaerobically. Aliquots were collected by centrifugation at indicated time points following incubation and the supernatant was decanted before the pellet was placed on dry ice and stored at −80°C.

### Examining bacterial abundance in the murine gut

Germ-free C57BL/6J (JAX # 000664) mice were bred and maintained in gnotobiotic isolators with a 12-hr light/dark cycle at Penn State University. All experiments were carried out using, 8–12-week-old mice with males and females at similar ratios. Experimental groups contained 5 mice and each group was provided with autoclaved standard mouse chow (5021, Lab Diet) or an irradiated glucose-sucrose chow (S4944, Bio-Serv) *ad libitum*. Diet information is available in supplemental tables S3 and S4, respectively. Animal gavage, fresh fecal sample collection, and relative strain abundance measurements were carried out as previously described.^16^ All experiments using mice were performed using protocols approved by the Penn State Institutional Animal Care and Use Committee

### Measuring BT4338 ortholog silencing by dietary sugar addition

*Bt* and *Bf* were grown as described above in 0.5% glucose, 0.5% fructose or 1.0% PMOG. Once mid-exponential phase growth was reached, a 1 ml sample was collected for downstream RNA preparation and a 20% glucose or fructose solution was added to PMOG grown cells to 0.2% final weight/volume and incubated for the indicated times. Subsequently, 1 ml samples were collected 10 and 60 min after the addition of either sugar solution, mRNA was harvested as described above, and transcript abundance was measured by qPCR using primers 1050 and 1051 to measure *Bt fusA2*, 1958, and 1959 to measure *Bf fusA2*, and 1956 and 1957 to measure universal 16s to normalize both genes.

### sRNA binding prediction using IntaRNA

In-silico analysis of putative sRNA interactions with the Roc mRNA leader was achieved using ThetaBase v2 to identify sRNAs upregulated during carbon limitation.^34^ Upregulated sRNAs were compared to BT4338 binding as predicted by ChIP-seq.^27^ Candidate sRNA binding analysis was performed using IntaRNA (v3.3.1) at default settings using the Vienna RNA package (2.5.0).^67^

### Statistical analysis

All data analysis was performed using Prism v9.3.1 (GraphPad, San Diego, California, USA). Western blot, ChIP, and qPCR experiments were conducted independently in at least biological triplicate. Data were expressed as mean ± standard error of the mean (SEM) and analyzed by one- or two-way ANOVA with Fisher’s Least Significant Difference test, or paired, two-tailed Student’s t-test where indicated and *P* values < 0.05 were statistically significant. Growth curves and *in vivo* bacterial abundance experiments were expressed as mean ± SEM with no additional statistical analysis conducted. Specific statistical tests, significance, and *n* are indicated in each figure legend.

## Supplementary Material

Supplemental Material

## Data Availability

The authors confirm that data supporting the findings in this study are available within the article and the indicated supplementary materials. Specific data points are openly available at doi: 10.17632/md44b9h2r5.1.
