# Supplementary material for: Dietary sugars silence the master regulator of carbohydrate utilization in human gut Bacteroides species
Source: Gut Microbes. 2023 Jun 26;15(1):2221484. doi: 10.1080/19490976.2023.2221484 (PMC10294740; doi:10.1080/19490976.2023.2221484)
Supplement: Supplemental Material [file KGMI_A_2221484_SM0656.docx]

**Supplementary Information**

**Dietary sugars silence the master regulator of carbohydrate utilization in human gut Bacteroides species**

Victoria H. Pearce^a,b,c^, Eduardo A. Groisman^d,e^ and Guy E. Townsend II^a,b,c*^

*^a^Penn State College of Medicine, Hershey, PA, USA*

*^b^Penn State Microbiome Center, Pennsylvania State University, State College, PA, USA*

*^c^Center for Molecular Carcinogenesis and Toxicology, Pennsylvania State University, State College, PA, USA*

*^d^Yale University School of Medicine, New Haven, CT, USA*

*^e^Microbial Sciences Institute, Yale University, New Haven, CT, USA*

*Address correspondence to Guy E. Townsend II: gtownsend@pennstatehealth.psu.edu; Department of Biochemistry and Molecular Biology, Penn State College of Medicine, 700 HMC Crescent Road, Hershey, Pennsylvania, USA

This file contains:

Figures S1 to S10

Tables S1 to S4

SI References

**
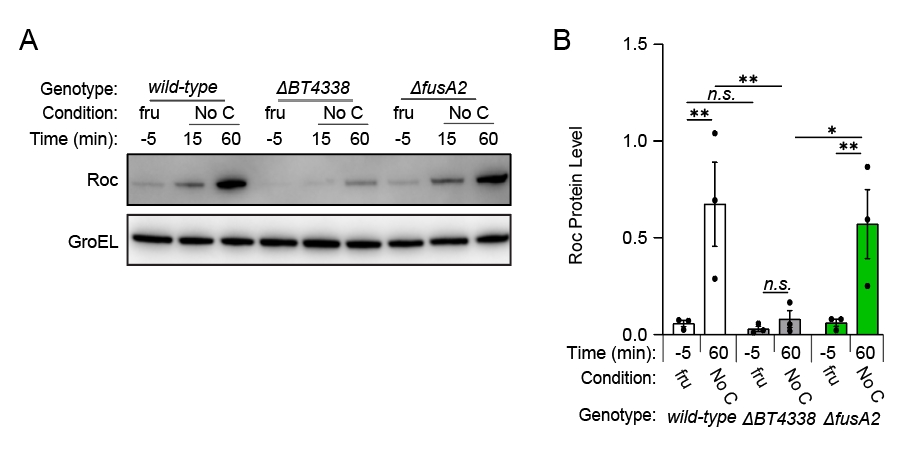
Figure S1.** BT4338 is required for Roc synthesis through carbon limitation. (a) Western blot analysis of Roc amounts from *wild-type* (GT593) or *Bt* strains deficient for *BT4338* (GT1234) or *fusA2* (GT1310) during growth in glucose (glu; -5) or 15-, and 60-minutes following exposure to carbon limitation (No C). Blot probed for anti-HA and anti-GroEL. (b) Quantified western blot analysis of Roc from strains described in (a) during growth in fructose (fru; -5) or 60-minutes following exposure to carbon limitation (No C) (*n* = 3 biological samples; error bars represent SEM, *P* values derived from two-way ANOVA; *n.s*. indicates *P* values ≥ 0.05; ** < 0.01).

**
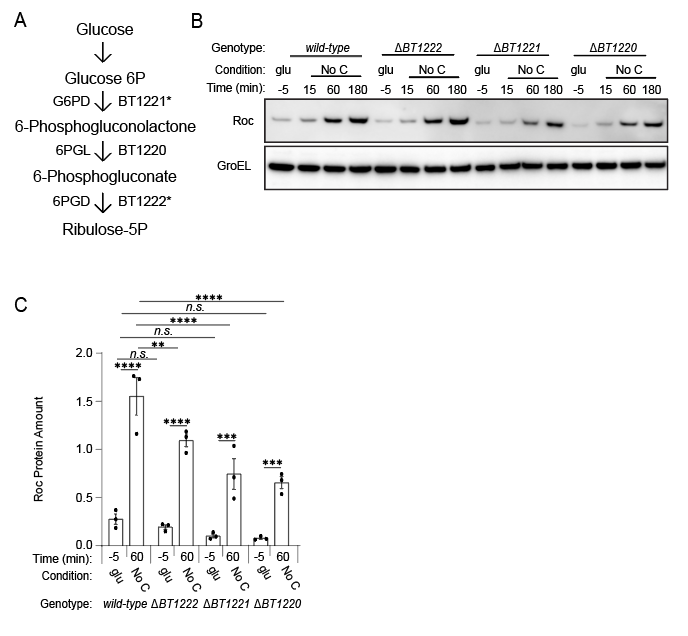
Figure S2.** Pentose phosphate pathway mutants exhibit reduced Roc amounts following carbon limitation. (a) Schematic of the oxidative pentose phosphate pathway with recovered loss-of-function mutants indicated with *. (b) Western blot analysis of Roc from *wild-type* *Bt* (GT426) or strains deficient for *BT1222* (GT2727), *BT1221* (GT2728), or *BT1220* (GT2729) during mid-exponential growth in glucose (glu; -5) or 15, 60, and 180-minutes following exposure to carbon limitation (No C). Blots were probed with anti-HA and anti-GroEL antibodies. (c) Quantified western blot analysis of strains described in (b) during mid-exponential growth in glucose (glu; -5) or 60-min following exposure to carbon limitation (No C) (*n* = 3 biological samples error; bars represent SEM, *P* values derived from two-way ANOVA; *n.s.* indicates *P* values ≥ 0.05; ***P* < 0.01; ****P* < 0.001; *****P* < 0.0001).

**
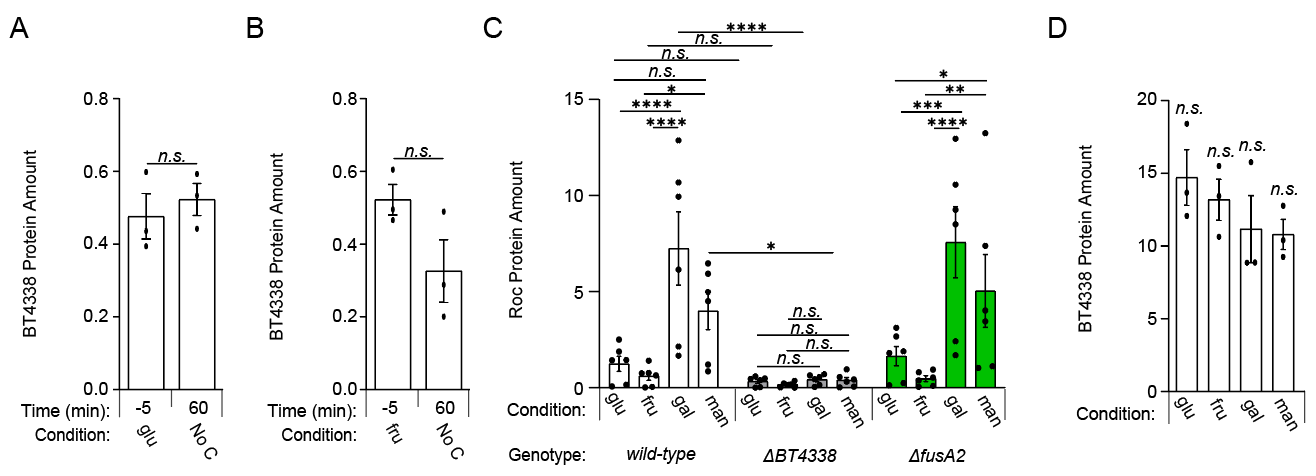
**

**Figure S3.** (a & b) Quantified western blot analysis of BT4338 from *Bt* strains expressing an epitope-tagged BT4338 (GT1481) during mid-exponential growth in (a) glucose (glu; -5) or (b) fructose (fru, -5) and after 60 minutes following exposure to carbon limitation (No C) from each condition (*n* = 3 biological samples; error bars represent SEM, *P* values derived from one-way ANOVA; *n.s.* indicates *P* values ≥ 0.05). (c) Quantified western blot analysis of *wild-type Bt* (GT593; white) or strains deficient for *BT4338* (GT1234; gray) or *fusA2* (GT1310; green) during mid-exponential growth in 0.5% glucose (glu), 0.5% fructose (fru), 0.5% galactose (gal), or 0.5% mannose (man) (*n* = 6 biological samples; error bars represent SEM, *P* values derived from two-way ANOVA; *n.s.* indicates *P* values ≥ 0.05; **P* < 0.05; ***P* < 0.01; ****P* < 0.001; *****P* < 0.0001). (d) Quantified western blot analysis of BT4338 from a *Bt* strain expressing an epitope-tagged BT4338 (GT1481) during mid-exponential growth during growth in 0.5% glucose (glu), 0.5% fructose (fru), 0.5% galactose (gal), or 0.5% mannose (man) (*n* = 3 biological samples; error bars represent SEM, *P* values derived from one-way ANOVA; *n.s.* indicates *P* values ≥ 0.05).

**
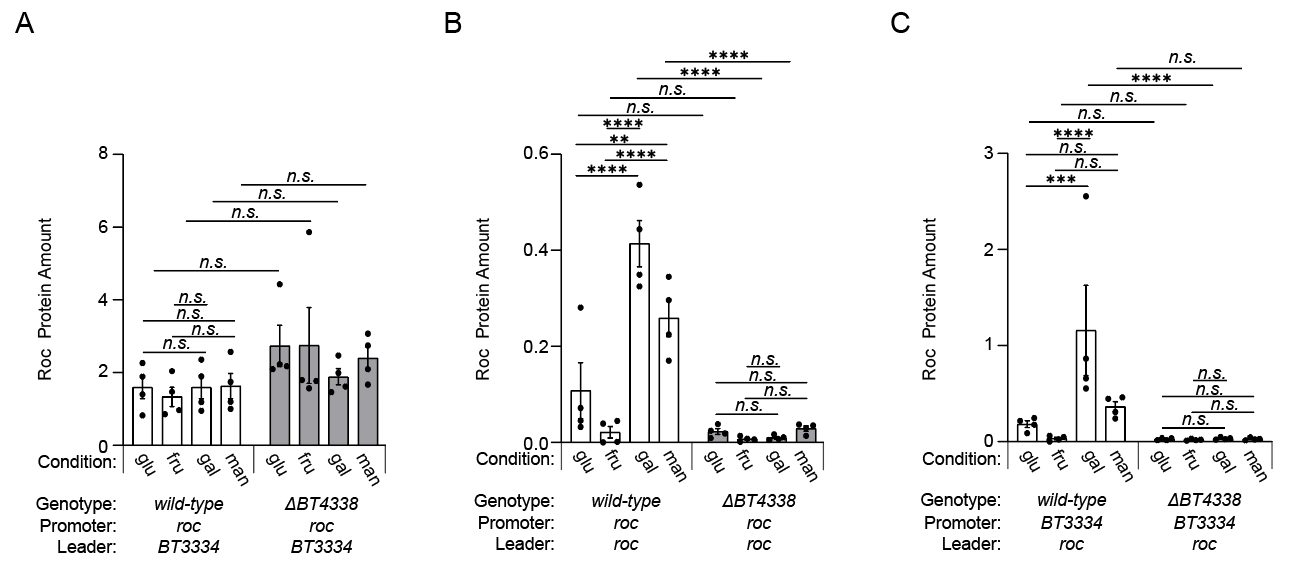
**
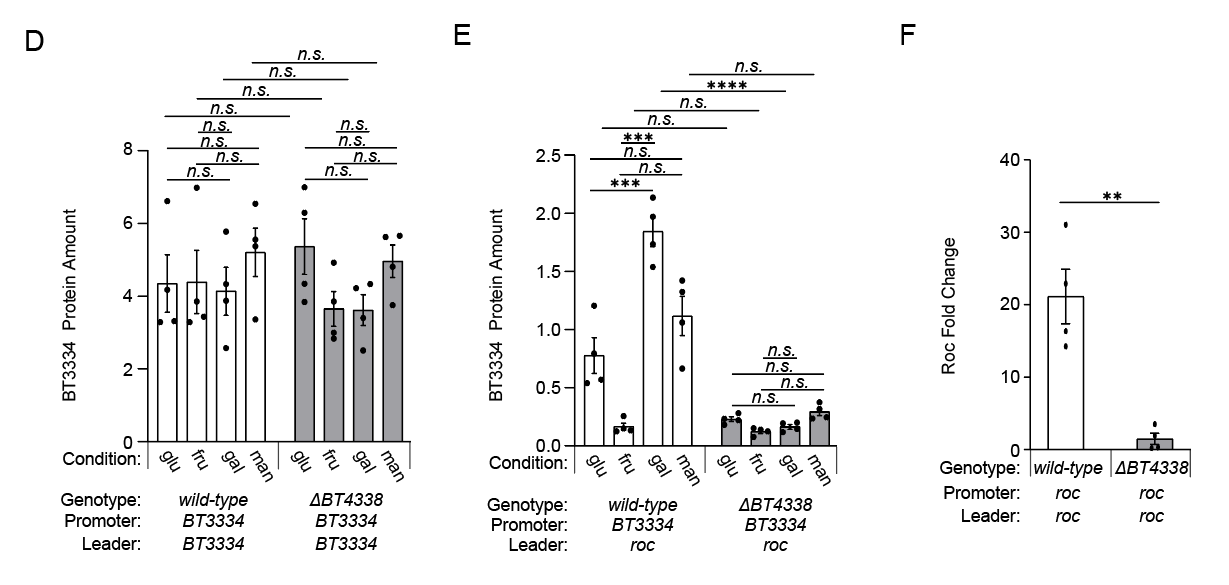

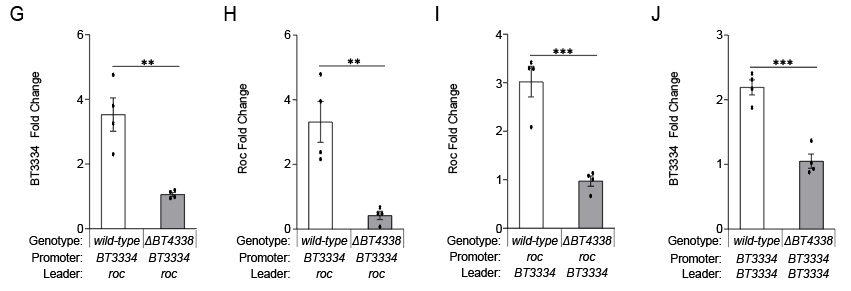


**Figure S4.** The *roc* mRNA leader is necessary and sufficient for *BT4338*-dependent control of the downstream ORF. (a - c) Quantified western blot analysis of Roc from engineered strains expressing the *roc* ORF positioned downstream of (a) the *roc* promoter and *BT3334* leader in backgrounds either encoding *BT4338* (GT665; white) or lacking *BT4338* (GT3510; gray), (b) the *roc* promoter and leader in backgrounds either encoding *BT4338* (GT530; white) or lacking *BT4338* (GT3509; gray), or (c) the *BT3334* promoter and the *roc* leader in backgrounds either encoding *BT4338* (GT670; white) or lacking *BT4338* (GT3511; gray) during mid-exponential growth in 0.5% glucose (glu), 0.5% fructose (fru), 0.5% galactose (gal), or 0.5% mannose (man) (*n* = 4 biological samples; error bars represent SEM; *P* values derived from two-way ANOVA; *n.s.* indicates *P* values ≥ 0.05; **P* < 0.05; ***P* < 0.01; ****P* < 0.001; *****P* < 0.0001). (d & e) Quantified western blot analysis of BT3334 from engineered strains expressing the *BT3334* ORF positioned downstream of (d) the *BT3334* promoter and leader in backgrounds either encoding *BT4338* (GT663; white) or lacking *BT4338* (GT3514; gray) or (e) the *BT3334* promoter and *roc* leader in backgrounds either encoding *BT4338* (GT534; white) or lacking *BT4338* (GT3512; gray) during mid-exponential growth in 0.5% glucose (glu), 0.5% fructose (fru), 0.5% galactose (gal), or 0.5% mannose (man) (*n* = 4 biological samples; error bars represent SEM; *P* values derived from two-way ANOVA; *n.s.* indicates *P* values ≥ 0.05; **P* < 0.05; ***P* < 0.01; ****P* < 0.001; *****P* < 0.0001). (f) Quantified western blot analysis depicting fold increase in Roc amounts from strains described in (b) following exposure to carbon limitation for 60-minutes (*n* = 4 biological samples; error bars represent SEM; *P* values derived from two-tailed t-test; ***P* < 0.01). (g) Quantified western blot analysis depicting the fold increase in BT3334 amounts from strains described in (e) following exposure to carbon limitation for 60-minutes. (h) Quantified western blot analysis depicting the fold increase in Roc amounts from strains described in (c) following exposure to carbon limitation for 60-minutes. (i) Quantified western blot analysis depicting the fold increase in Roc amounts from strains described in (a) following exposure to carbon limitation for 60-minutes. (j) Quantified western blot analysis depicting the fold increase in BT3334 amounts from strains described in (d) following exposure to carbon limitation for 60-minutes (for panels (f – j), *n* = 4 biological samples; error bars represent SEM; *P* values derived from two-tailed t-test; ***P* < 0.01; ****P* < 0.001).

**
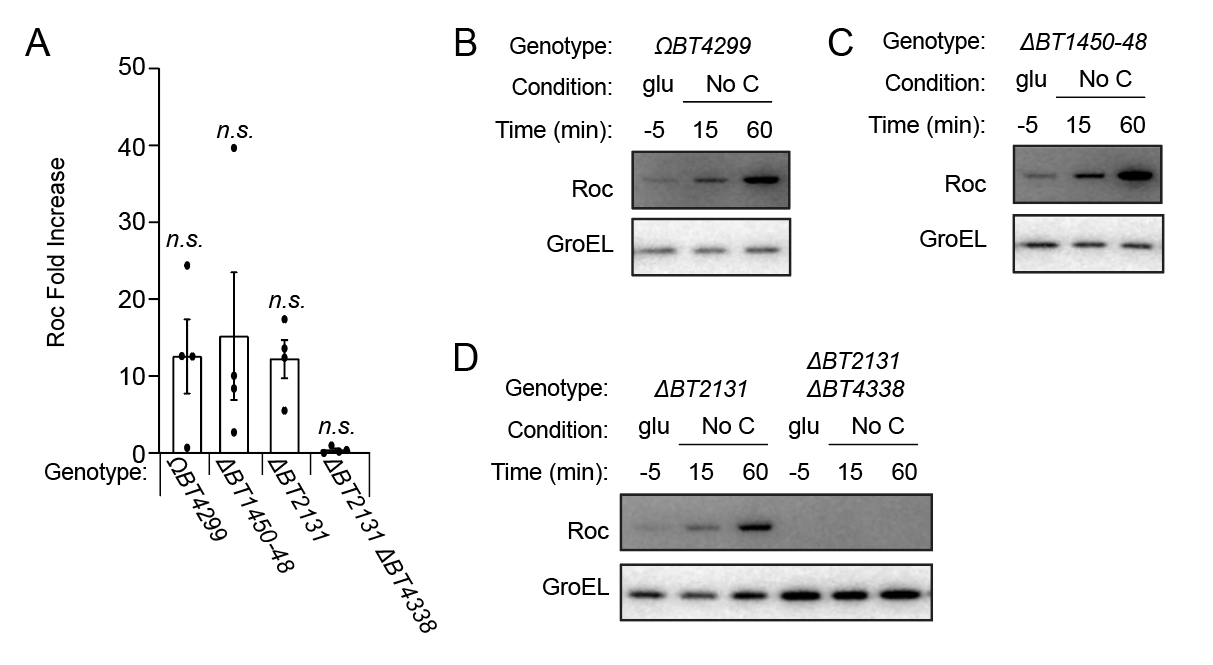
Figure S5.** Candidate BT4338 regulated products are not required for Roc synthesis. (a) Quantified western blot analysis of strains deficient for *BT4299* (GT1459), *BT1450-48* (GT1427), or *BT2131* (GT1372), or both *BT2131* and *BT4338* (GT4361). Bars represent the fold increase between (glu; -5) and 60-minute time points (*n* = 4 biological samples; error bars represent SEM; *P* values derived from one-way ANOVA calculated against isogenic *wild-type* GT593 or GT1234 for the *BT4338* deficient strain; *n.s.* indicates *P* values ≥ 0.05). (b - d) Western blot analysis of Roc levels from strains deficient for (b) *BT4299* (GT1459), (c) *BT1450-48* (GT1427), or (d) *BT2131* (GT1372) and both *BT2131* and *BT4338* (GT1374). Blots were probed using anti-HA and anti-GroEL antibodies.

**
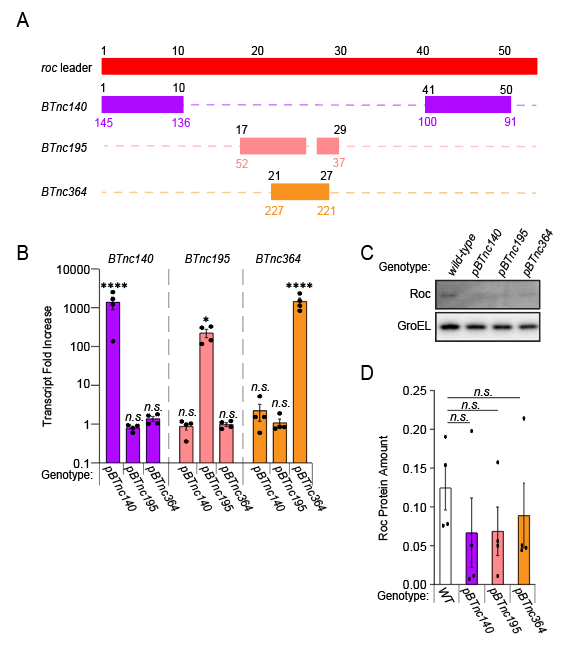
Figure S6.** Expression of candidate sRNAs are insufficient to increase Roc amounts. (a) Schematic of selected sRNAs *BTnc140* (purple), *BTnc195* (pink), *BTnc364* (orange) complementarity to the *roc* leader (red). (b) The transcript amounts of *BTnc140* (purple), *BTnc195* (pink), or *BTnc364* (orange) were measured in strains overexpressing each of these sRNAs during mid-exponential growth in 0.5% glucose compared to *wild-type Bt* (GT593) (*n* = 4 biological replicates; error bars represent SEM; *P* values derived from two-way ANOVA; *n.s.* indicates P values ≥ 0.05; **P* < 0.05; *****P* < 0.0001). (c) Western blot analysis of Roc amounts from *wild-type* *Bt* (GT593) or strains heterologously expressing *BTnc140* (GT4340), *BTnc195* (GT4344), or *BTnc364* (GT4381) during mid-exponential growth in glucose. Blots were probed using anti-HA and anti-GroEL antibodies. (d) Quantified western blot analysis of strains described in (c) (*n* = 4 biological samples; error bars represent SEM; *P* values derived from one-way ANOVA; *n.s.* indicates *P* values ≥ 0.05).

**
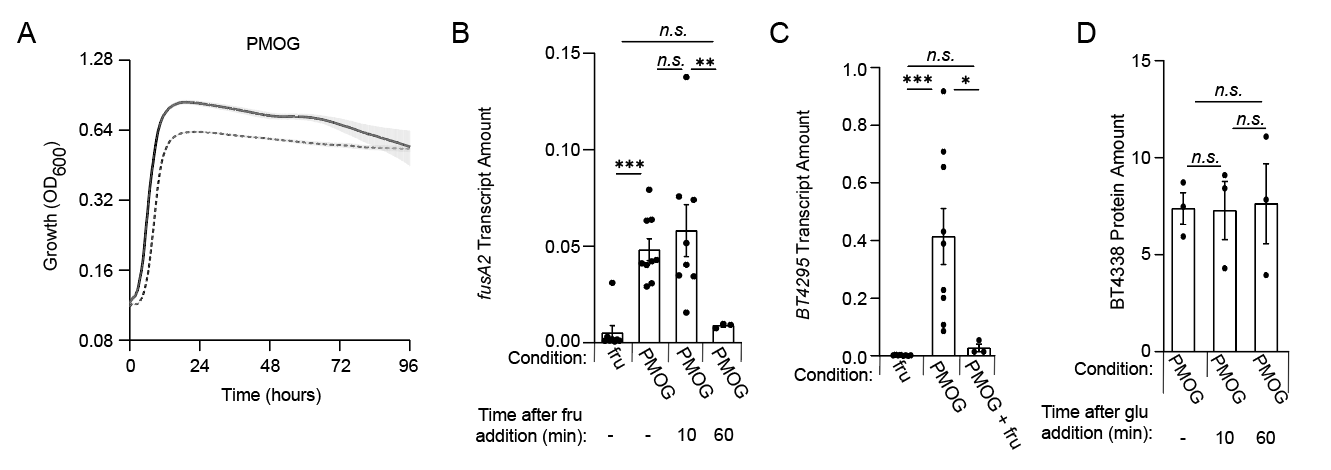
Figure S7.** *BT4338* is dispensable for growth in PMOG and necessary for *fusA2* and *BT4295* expression. (a) Growth of *wild-type* (ATCC 29148; solid line) or isogenic *BT4338*-deficient (GT2623; dotted line) *Bt* strains in minimal media containing 1% PMOG (*n* = 4 biological samples; error bars represent SEM). (b) qPCR analysis of *fusA2* (*BT2167*) transcript amounts measured in *wild-type Bt* (ATCC 29148) during mid-exponential growth in either 0.5% fructose (fru) or 1% PMOG, and 10 and 60-minutes following the addition of fructose to 0.2% (*n* = 9 biological samples for fru, PMOG, and PMOG + fru 10m; *n* = 3 for PMOG + fru 60m; error bars represent SEM, *P* values derived from one way ANOVA; *n.s.* indicates *P* values ≥ 0.05; ***P* < 0.01; ****P* < 0.001). (c) qPCR analysis of *BT4295* transcript amounts measured in *wild-type Bt* (ATCC 29148) during mid-exponential growth in either 0.5% fructose (fru) or 1% PMOG, and 60-minutes following the addition of fructose to 0.2% (n = 9 biological samples for fru and PMOG; n = 3 for PMOG + fru; error bars represent SEM, *P* values derived from one way ANOVA; *n.s.* indicates *P* values ≥ 0.05; **P* < 0.05; ****P* < 0.001). (d) Quantified western blot analysis of BT4338 protein amounts from a *Bt* strain expressing epitope-tagged BT4338 (GT1481) during mid-exponential growth in 1% PMOG, and 10 and 60-minutes following the addition of glucose to 0.2% (*n* = 3 biological samples; error bars represent SEM, *P* values derived from two-way ANOVA; *n.s.* indicates *P* values ≥ 0.05).

**
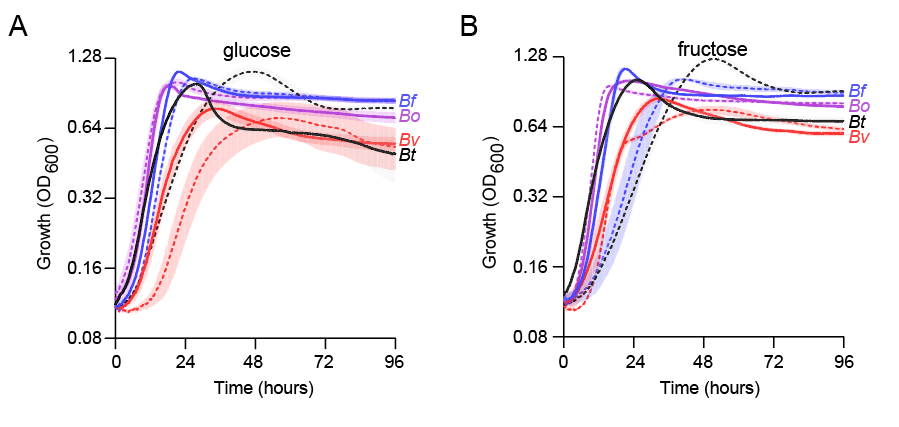
Figure S8.** BT4338 orthologs are dispensable for growth in glucose and fructose across Bacteroides species. (a – b) Growth of bar-coded *wild-type* (solid lines) *B. thetaiotaomicron* (*Bt*; GT3361; black), *B. fragilis* (*Bf*; GT3551; blue), *B. vulgatus* (*Bv*; GT3367; red), or *B. ovatus* (*Bo*; GT3364; purple) or bar-coded isogenic *BT4338*-ortholog-deficient strains (dashed lines; GT3522, GT3555, GT3643, GT3553, respectively) in minimal media containing 0.5% (a) glucose or (b) fructose as the sole carbon source (*n* = 4 biological replicates; error bars represent SEM).

**
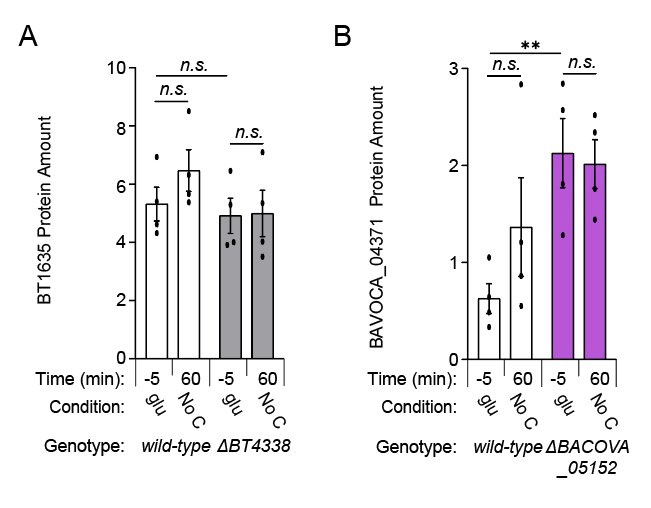
**

**Figure S9.** Roc homologs are differentially regulated by BT4338 orthologs. (a) Quantified western blot analysis of BT1635 protein amounts from *wild-type* *Bt* (GT4372; white) or a strain deficient for *BT4338* (GT4373; gray) (*n* = 4 biological samples; error bars represent SEM; *P* values derived from one-way ANOVA; *n.s.* indicates *P* values ≥ 0.05). (b) Quantified western blot analysis of BACOVA_04371 amounts from *wild-type* *Bo* (GT4362; white) or a strain deficient for *BACVOA_05152* (GT4369; purple) (*n* = 4 biological samples; error bars represent SEM; *P* values derived from one-way ANOVA; *n.s.* indicates *P* values ≥ 0.05; ***P* < 0.01).


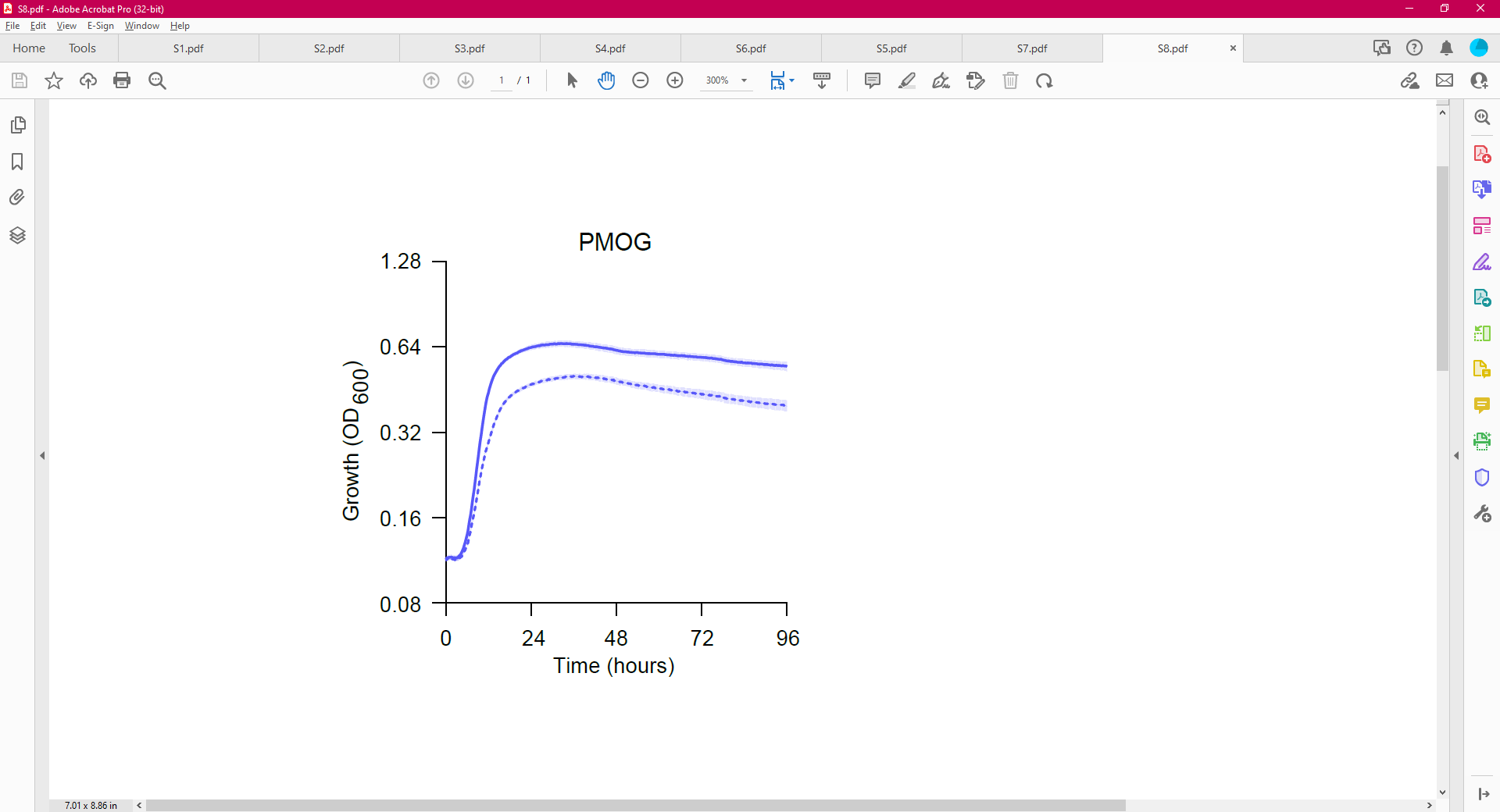
**Figure S10.** Growth of *wild-type* *B. fragilis* (ATCC 25285; solid line) or an isogenic *BT4338*-ortholog-deficient strain (GT2520; dashed line) in minimal media containing 1% PMOG (*n* = 4 biological replicates; error bars represent SEM).

| Table S1. Strains and plasmids used in this study | | | |
| --- | --- | --- | --- |
| **Identifier** | ***Description*** | **Genotype** | **Source** |
| ***B. thetaiotaomicron*** | | | |
| ATCC 29148 | *wild-type Bt* |  | ATCC |
| GT23 | *A tdk-deficient strain* | Δ*tdk* | ^[1]^ |
| GT593 | *A strain harboring an in-frame C-terminally HA-tagged roc* | *∆tdk BT3172-HA* | ^[2]^ |
| GT1234 | *A cur-deficient strain harboring an in-frame C-terminally HA-tagged roc* | *∆tdk BT3172-HA ΔBT4338* | This study |
| GT1310 | *A fusA2-deficient strain harboring an in-frame C-terminally HA-tagged roc* | *∆tdk BT3172-HA ΔBT2167* | This study |
| GT1663 | *A strain harboring a chromosomal roc deletion and a multi-copy plasmid encoding C-terminally HA-tagged roc* | *∆tdk ∆BT3172 + pLYL01::BT3172-HA* | This study |
| GT3148 | *A GT1663-derived strain harboring a transposon insertion in BT1222* | *∆tdk ∆BT3172 BT1222::Tn + pLYL01::BT3172-HA* | This study |
| GT3150 | *A GT1663-derived strain harboring a transposon insertion in BT1221* | *∆tdk ∆BT3172 BT1221::Tn + pLYL01::BT3172-HA* | This study |
| GT3151 | *A GT1663-derived strain harboring a transposon insertion in BT4338* | *∆tdk ∆BT3172 BT4338::Tn + pLYL01::BT3172-HA* | This study |
| GT1481 | *A strain expressing a C-terminally HA-tagged BT4338 gene* | *∆tdk ∆BT4338 att-1::pNBU2-tetQ-PBT4338-4xG-HA* | ^[3]^ |
| GT426 | *A strain expressing a C-terminally HA-tagged roc gene* | *∆tdk pKNOCK-tetQ::BT3172-HA* | ^[2]^ |
| GT530 | *A strain harboring chromosomal deletions of roc and BT3334 complemented with a single-copy plasmid encoding C-terminally HA-tagged roc expressed from its native promoter and leader* | *∆tdk ∆BT3334 ∆BT3172 att-1::pNBU2-tetQ-BT3172-HA* | ^[2]^ |
| GT534 | *A strain harboring chromosomal deletions of roc and BT3334 complemented with a single-copy plasmid encoding C-terminally HA-tagged BT3334 expressed from its native promoter and leader* | *∆tdk ∆BT3334 ∆BT3172 att-1::pNBU2-tetQ-BT3334-HA* | ^[2]^ |
| GT640 | *A strain harboring chromosomal deletions of roc and BT3334 complemented with a single-copy plasmid encoding C-terminally HA-tagged BT3334 expressed from the roc promoter and native leader* | *∆tdk ∆BT3334 ∆BT3172 att-1::pNBU2-tetQ-P_BT3172_-BT3334LEADER-BT3334-HA* | ^[2]^ |
| GT663 | *A strain harboring chromosomal deletions of roc and BT3334 complemented with a single-copy plasmid encoding C-terminally HA-tagged BT3334 expressed from its native promoter and the roc leader* | *∆tdk ∆BT3334 ∆BT3172 att-1::pNBU2-tetQ-P_BT3334_-BT3172LEADER-BT3334-HA* | ^[4]^ |
| GT665 | *A strain harboring chromosomal deletions of roc and BT3334 complemented with a single-copy plasmid encoding C-terminally HA-tagged roc expressed from its native promoter and the BT3334 leader* | *∆tdk ∆BT3334 ∆BT3172 att-1::pNBU2-tetQ-P_BT3172_-BT3334LEADER-BT3172-HA* | ^[4]^ |
| GT670 | *A strain harboring chromosomal deletions of roc and BT3334 complemented with a single-copy plasmid encoding C-terminally HA-tagged roc expressed from the BT3334 promoter and its native leader* | *∆tdk ∆BT3334 ∆BT3172 att-1::pNBU2-tetQ-P_BT3334_-BT3172LEADER-BT3172-HA* | ^[4]^ |
| GT2623 | *A cur-deficient strain* | *ΔBT4338* | This study |
| GT2727 | *a strain lacking BT1222 expressing a C-terminally HA-tagged roc gene* | *∆tdk ΔBT1222 pKNOCK-tetQ::BT3172-HA* | This study |
| GT2728 | *a strain lacking BT1221 expressing a C-terminally HA-tagged roc gene* | *∆tdk ΔBT1221 pKNOCK-tetQ::BT3172-HA* | This study |
| GT2729 | *a strain lacking BT1220 expressing a C-terminally HA-tagged roc gene* | *∆tdk ΔBT1220 pKNOCK-tetQ::BT3172-HA* | This study |
| GT3509 | *A strain harboring chromosomal deletions of roc, BT3334, and cur complemented with a single-copy plasmid encoding C-terminally HA-tagged roc expressed from its native promoter and leader* | *∆tdk ∆BT3172 ∆BT3334 ∆BT4338 att-1::pNBU2-tetQ-BT3172-HA* | This study |
| GT3510 | *A strain harboring chromosomal deletions of roc, BT3334, and cur complemented with a single-copy plasmid encoding C-terminally HA-tagged roc expressed from its native promoter and the BT3334 leader* | *∆tdk ∆BT3172 ∆BT3334 ∆BT4338 att-1::pNBU2-tetQ-P_BT3172_-BT3334LEADER-BT3172-HA* | This study |
| GT3511 | *A strain harboring chromosomal deletions of roc, BT3334, and cur complemented with a single-copy plasmid encoding C-terminally HA-tagged roc expressed from the BT3334 promoter and its native leader* | *∆tdk ∆BT3172 ∆BT3334 ∆BT4338 att-1::pNBU2-tetQ-P_BT3334_-BT3172LEADER-BT3172-HA* | This study |
| GT3512 | *A strain harboring chromosomal deletions of roc, BT3334, and cur complemented with a single-copy plasmid encoding C-terminally HA-tagged BT3334 expressed from its native promoter and leader* | *∆tdk ∆BT3172 ∆BT3334 ∆BT4338 att-1::pNBU2-tetQ-BT3334-HA* | This study |
| GT3513 | *A strain harboring chromosomal deletions of roc, BT3334, and cur complemented with a single-copy plasmid encoding C-terminally HA-tagged BT3334 expressed from the roc promoter and its native leader* | *∆tdk ∆BT3172 ∆BT3334 ∆BT4338 att-1::pNBU2-tetQ-P_BT3172_-BT3334LEADER-BT3334-HA* | This study |
| GT3514 | *A strain harboring chromosomal deletions of roc, BT3334, and cur complemented with a single-copy plasmid encoding C-terminally HA-tagged BT3334 expressed from its native promoter and the roc leader* | *∆tdk ∆BT3172 ∆BT3334 ∆BT4338 att-1::pNBU2-tetQ-P_BT3334_-BT3172LEADER-BT3334-HA* | This study |
| GT3361 | *a bar-coded wild-type Bt strain* | *att-1::pNBU2-tetQ-BC01* | This study |
| GT3522 | *a bar-coded cur-deficient strain* | *ΔBT4338 att-1::pNBU2-tetQ-BC06* | This study |
| GT3363 | *a bar-coded strain lacking the native cur gene complemented with cur* | *ΔBT4338 att-1::pNBU2-tetQ-BC04-BT4338* | This study |
| GT1372 | *A strain harboring a chromosomal deletion of BT2131 and an in-frame C-terminally HA-tagged roc* | *Δtdk BT3172-HA ΔBT2131* | This study |
| GT1374 | *A strain harboring a chromosomal deletion of BT2131 and cur with an in-frame C-terminally HA-tagged roc* | *Δtdk BT3172-HA ΔBT2131 ΔBT4338* | This study |
| GT1427 | *A strain harboring a chromosomal deletion of BT1450-48 and an in-frame C-terminally HA-tagged roc* | *Δtdk BT3172-HA ΔBT1450-48* | This study |
| GT1459 | *A strain harboring a BT4299 insertion and an in-frame C-terminally HA-tagged roc* | *Δtdk BT3172-HA pKNOCK-tetQ::BT4299* | This study |
| GT4358 | *A strain harboring an in-frame C-terminally HA-tagged roc* | *pKNOCK-tetQ::BT3172-HA* | This study |
| GT4359 | *A strain harboring a chromosomal deletion of cur and an in-frame C-terminally HA-tagged roc* | *pKNOCK-tetQ::BT3172-HA ΔBT4338* | This study |
| GT4372 | *A strain harboring an in-frame C-terminally HA-tagged BT1635* | *pKNOCK-tetQ::BT1635-HA* | This study |
| GT4373 | *A strain harboring a chromosomal deletion of cur and an in-frame C-terminally HA-tagged BT1635* | *pKNOCK-tetQ::BT1635-HA ΔBT4338* | This study |
| GT4340 | *A strain harboring an in-frame C-terminally HA-tagged roc and a multi-copy plasmid encoding BTnc140 following the 16S promoter* | *Δtdk BT3172-HA pLYL01-tet::P-16S-nc140* | This study |
| GT4344 | *A strain harboring an in-frame C-terminally HA-tagged roc and a multi-copy plasmid encoding BTnc195 following the 16S promoter* | *Δtdk BT3172-HA pLYL01-tet::P-16S-nc195* | This study |
| GT4381 | *A strain harboring an in-frame C-terminally HA-tagged roc and a multi-copy plasmid encoding BTnc364 following the 16S promoter* | *Δtdk BT3172-HA pLYL01-tet::P-16S-nc364* | This study |
| ***B. fragilis*** | | | |
| ATCC 25285 | *Wild-type Bf* |  | ATCC |
| GT2520 | a *cur*-deficient strain | *ΔBF9343_0915* | This study |
| GT3551 | a bar-coded wild-type *Bf* strain | att-1::pNBU2-tetQ-BC01 | This study |
| GT3555 | a bar-coded *cur*-deficient strain | *ΔBF9343_0915* att-1::pNBU2-tetR-BC06 | This study |
| ***B. ovatus*** | | | |
| ATCC 8483 | *Wild-type Bo* |  | ATCC |
| GT2413 | *a cur-deficient strain* | *ΔBACOVA_05152* | This study |
| GT3364 | *a bar-coded wild-type Bo strain* | *att-1::pNBU2-tetQ-BC01* | This study |
| GT3553 | *a bar-coded cur-deficient strain* | *ΔBACOVA_05152 att-1:: pNBU2-tetQ-BC06* | This study |
| GT4362 | *A strain harboring an in-frame C-terminally HA-tagged BACOVA_04371* | *pKNOCK-tetQ:: BACOVA_04371-HA* | This study |
| GT4369 | *A strain harboring a chromosomal deletion of cur and an in-frame C-terminally HA-tagged BACOVA_04371* | *pKNOCK-tetQ:: BACOVA_04371-HA ΔBACOVA_05152* | This study |
| ***B. vulgatus*** | | | |
| ATCC 8482 | *Wild-type Bv* |  | ATCC |
| GT2399 | *a cur-deficient strain* | *ΔBVU_3580* | This study |
| GT3367 | *a bar-coded wild-type Bv strain* | *att-1::pNBU2-tetQ-BC01* | This study |
| GT3643 | *a bar-coded cur-deficient strain* | *ΔBVU_3580 att-1:: pNBU2-tetQ-BC06* | This study |
| ***E. coli*** | | | |
| GT1 | *S17-1* | λpir | ^[5]^ |
| **Plasmids** | | | |
| **Identifier** | ***Description*** | **Source** |  |
| *pSAM-Bt* | A plasmid-borne mariner transposon | ^[6]^ |  |
| *pEXCHANGE-tdk* | A plasmid for engineering chromosomal gene deletions in *tdk*-deficient *Bt* strains | ^[1]^ |  |
| *pEXCHANGE-tdk-ΔBT1222* | A plasmid used to delete *BT1222* | This study |  |
| *pEXCHANGE-tdk-ΔBT1221* | A plasmid used to delete *BT1221* | This study |  |
| *pEXCHANGE-tdk-ΔBT1220* | A plasmid used to delete *BT1220* | This study |  |
| *pSIE1* | A plasmid for engineering chromosomal deletion in Bacteroides species | ^[7]^ |  |
| *pSIE1-ΔBT4338* | A plasmid used to delete cur from the *Bt* chromosome | This study |  |
| *pSIE1-ΔBvu_3580* | A plasmid used to delete cur from the *Bv* chromosome | This study |  |
| *pSIE1-ΔBACOVA_05152* | A plasmid used to delete cur from the *Bo* chromosome | This study |  |
| *pLGB13* | A plasmid for engineering chromosomal deletion in Bacteroides species | ^[8]^ |  |
| *pLGB13-ΔBF9343_0915* | A plasmid used to delete cur from the *Bf* chromosome | This study |  |
| *pRK231* | A helper plasmid for conjugation into *Bf* | ^[9]^ |  |
| *pNBU2-tetQ-BC01* | A plasmid bearing a unique molecular bar-code | ^[2]^ |  |
| *pNBU2-tetQ-BC04* | A plasmid bearing a unique molecular bar-code | ^[2]^ |  |
| *pNBU2-tetQ-BC06* | A plasmid bearing a unique molecular bar-code | ^[2]^ |  |
| *pLYL01-BT3172-HA* | A multi-copy plasmid expressing a C-terminally HA-tagged *roc* gene from its native promoter and leader | ^[2]^ |  |
| *pNBU2-tetQ-BT3172-HA* | A single-copy plasmid expressing a C-terminally HA-tagged *roc* gene from its native promoter and leader | ^[2]^ |  |
| *pNBU2-tetQ-PBT3172-BT3334LEADER-BT3172-HA* | A single-copy plasmid expressing a C-terminally HA-tagged *roc* gene from its native promoter and the *BT3334* leader | ^[2]^ |  |
| *pNBU2-tetQ-PBT3334-BT3172LEADER-BT3172-HA* | A single-copy plasmid expressing a C-terminally HA-tagged *roc* gene from the *BT3334* promoter and its native leader | ^[2]^ |  |
| *pNBU2-tetQ-BT3334-HA* | A single-copy plasmid expressing a C-terminally HA-tagged *BT3334* gene from its native promoter and leader | ^[2]^ |  |
| *pNBU2-tetQ-PBT3172-BT3334LEADER-BT3334-HA* | A single-copy plasmid expressing a C-terminally HA-tagged *BT3334* gene from the *roc* promoter and its native leader | ^[2]^ |  |
| *pNBU2-tetQ-PBT3334-BT3172LEADER-BT3334-HA* | A single-copy plasmid expressing a C-terminally HA-tagged *BT3334* gene from its native promoter and the *roc* leader | ^[2]^ |  |
| *pKNOCK-tetQ-BT3172-HA* | A plasmid that C-terminally HA-tags *roc* in its chromosomal locus | ^[2]^ |  |
| *pKNOCK-tetQ::BT4299* | A plasmid that inactivates *BT4299* and downstream genes. | This study |  |
| *pKNOCK-tetQ:: BACOVA_04371-HA* | A plasmid that C-terminally HA-tags *BACOVA_04371* in its chromosomal locus | This study |  |
| *pKNOCK-tetQ::BT1635-HA* | A plasmid that C-terminally HA-tags *BT1635* in its chromosomal locus | ^[2]^ |  |
| *pLYL01-tet::P-16S-nc140* | A multi-copy plasmid expressing *BTnc140* from the *Bt* 16s promoter. | This study |  |
| *pLYL01-tet::P-16S-nc195* | A multi-copy plasmid expressing *BTnc195* from the *Bt* 16s promoter. | This study |  |
| *pLYL01-tet::P-16S-nc364* | A multi-copy plasmid expressing *BTnc364* from the 16s promoter. | This study |  |

| Table S2. Oligonucleotides used in this study | | | | |
| --- | --- | --- | --- | --- |
| **ID** | **Name** | **Sequence** | **Purpose** | **Source** |
| **qPCR** | | | | |
| 1044 | qBt16sF | GGTAGTCCACACAGTAAACGATGAA | Measuring *16s* rRNA transcript levels from *Bt* by qPCR | ^[2]^ |
| 1045 | qBt16sR | CCCGTCAAATTCCTTTGAGTTTC |  | ^[2]^ |
| 1050 | qBT2167f | AAAACGTCGCGGATCTGTTG | Measuring *fusA2* transcript levels from *Bt* by qPCR | ^[3]^ |
| 1051 | qBT2167r | TGGAGAACGGTAGAGAAAACGG |  | ^[3]^ |
| 1995 | ChIP_roc_f | AGAAGGGCAACTGGACAAAG | Measuring roc promoter levels by qPCR following ChIP | This study |
| 1996 | ChIP_roc_r | CCTTTCACGGTGCTGAATGAG |  | This study |
| 1997 | ChIP_fusA2_f | ATAAGTCTGGCCTGTCTGCTAC | Measuring *fusA2* promoter levels by qPCR following ChIP | ^[3]^ |
| 1998 | ChIP_fusA2_r | AGGGATTTATTGGGGGAAAAGC |  | ^[3]^ |
| 1999 | ChIP_rpoD_f | GTCAGTGATCTGGAAGAAGCAATG | measuring *rpoD* promoter levels by qPCR following ChIP | ^[3]^ |
| 2000 | ChIP_rpoD_r | GGGAATACACCTGTCAGGAACAA |  | ^[3]^ |
| 1956 | qBacteroides_16Sf | TGAAAGTTTGCGGCTCAACC | Measuring *16s* rRNA transcript levels from Bacteroides species by qPCR | This study |
| 1957 | qBacteroides_16Sr | AAGCATTTCACCGCTACACC |  | This study |
| 1958 | qBF9343_fusA2f | AGAAGACGAAATGCGCGAAG | measuring *fusA2* transcript levels from *Bf* by qPCR | This study |
| 1959 | qBF9343_fusA2r | ATCTTTGCCTGCACATACGC |  | This study |
| 1962 | qBACOVA_fusA2r | TGGAGAACGGTGGAGAAAACG | measuring *fusA2* transcript levels from *Bo* by qPCR | This study |
| 1965 | qBVU_fusA2f | AATGTGCTCGAACAGTTGCG | Measuring *fusA2* transcript levels from *Bf* by qPCR | This study |
| 1966 | qBVU_fusA2r | AGGTCCCGTTTCAAGAGGATAC |  | This study |
| 2028 | pNBU2_tet_BC01 | ATGTCGCCAATTGTCACTTTCTCA | Measuring relative strain abundance from mouse fecal pellets | ^[2]^ |
| 2030 | pNBU2_tet_BC04 | CTCCATAAAGGCGCATACCGACTA |  | ^[2]^ |
| 2031 | pNBU2_tet_BC06 | GATTACGGCGTGATAGATTGGTGT |  | ^[2]^ |
| 2033 | UNIV-R | CACAATATGAGCAACAAGGAATCC |  | ^[2]^ |
| 2676 | qBTnc140f | AGCAGGTTTGTACCCTCTAAGG | Measuring *BTnc140* rRNA transcript levels from *Bt* by qPCR | This study |
| 2677 | qBTnc140r | TTTCGGTTAGGCTCTGGTAAGC |  | This study |
| 2678 | qBTnc195f | ACCACAGGTTACACAGTTTTGC | Measuring *BTnc195* rRNA transcript levels from *Bt* by qPCR | This study |
| 2679 | qBTnc195r | CTCGTCAGCATGGATATTCACC |  | This study |
| 2680 | qBTnc364f | TCTGATACTCGGCACCAGAAAG | Measuring *BTnc364* rRNA transcript levels from *Bt* by qPCR | This study |
| 2681 | qBTnc364r | AGATAGAAGCCATCCAGTGCAG |  | This study |
|  | ***Tn insertion site identification*** | | | |
| 1064 | AR1A | GGCCACGCGTCGACTAGTACNNNNNNNNNNGTAAT | Semi-random PCR for determining sites of transposon insertion | ^[6]^ |
| 1065 | AR1B | GGCCACGCGTCGACTAGTACNNNNNNNNNNGATGC |  | ^[6]^ |
| 1066 | AR1C | GGCCACGCGTCGACTAGTACNNNNNNNNNNGGCCG |  | ^[6]^ |
| 1067 | AR1D | GGCCACGCGTCGACTAGTACNWNWNWNWNWCTTAA |  | ^[6]^ |
| 1068 | AR2 | GGCCACGCGTCGACTAGTAC |  | ^[6]^ |
| 1069 | PATseq1 | ACGTACTCATGGTTCATCCCGATA |  | ^[6]^ |
| 1070 | PATseq2 | GCGTATCGGTCTGTATATCAGCAA |  | ^[6]^ |
|  | ***Plasmid construction*** | | | |
| 1459 | pEXCHANGE-dBT1222_5f | GCTCTAGAACTAGTGGATCCAGGAAGATATGCAACAGAAAGATAACTC | Constructing a plasmid to delete *BT1222* from the *Bt* genome | This study |
| 1460 | dBT1222_5r | ATCTTATATATTTTAAAATATTGTTTATGTAAGAAACAC |  | This study |
| 1461 | dBT1222_3f | TAAAATATATAAGATGTACTTTTCTTTTGCCTTGATGCAAAAG |  | This study |
| 1462 | pEXCHANGE-dBT1222_3r | AGATAACATTCGAGTCGACGCCCGGCCGCCTTCAAAT |  | This study |
| 1463 | pEXCHANGE-dBT1221_5f | GCTCTAGAACTAGTGGATCCATGGAATCTGGATTTGGCTTCCA | Constructing a plasmid to delete *BT1221* from the *Bt* genome | This study |
| 1464 | dBT1221_5r | CGTCGTATTTTTAACTTTTTACCTTTTATCC |  | This study |
| 1465 | dBT1221_3f | GTTAAAAATACGACGATGGAGACAGCCCGTTCGTTG |  | This study |
| 1466 | pEXCHANGE-dBT1221_3r | AGATAACATTCGAGTCGACCCAATGTTACTCTCTCTTCTTATATATAAT |  | This study |
| 1467 | pEXCHANGE-dBT1220_5f | GCTCTAGAACTAGTGGATCCGGATAATTTCCGGAATGAGGTAGTG | Constructing a plasmid to delete *BT1220* from the *Bt* genome | This study |
| 1468 | dBT1220_5r | ATTGTCTCTCTCTTTAGACGACGGGAAAACAGATAGTT |  | This study |
| 1469 | dBT1220_3f | AAAGAGAGAGACAATATAAAGACAAAGTATATTATA |  | This study |
| 1470 | pEXCHANGE-dBT1220_3r | AGATAACATTCGAGTCGACACAAACACATAACTGCCTCCTGC |  | This study |
| 1612 | pLGB13-dBF9343_0915_5f | GATTAGCATTATGAGGATCCAGCTATCTAACAGAAAGCCCTTAGAAATTACTAC | Constructing a plasmid to delete *cur* from the *Bf* genome | This study |
| 1613 | dBF9343_0915_5r | ATCTGTTGCAAAACTGTGTTATAAAGCACA |  | This study |
| 1691 | dBF9343_0915_3f: | ACACAGTTTTGCAACAGATAAGCGCTTTGTCGCCATTCA |  | This study |
| 1617 | pLGB13-dBF9343_0915_3r | TCCACCGCGGTGGCGGCCGCTGATGTGGTCGTTAATATACTTCTATTTCGTC |  | This study |
| 1618 | pSIE1-dBvu_3580_5f | GATTAGCATTATGAGGATCCTGAAGGTGACGGCTACTCG | Constructing a plasmid to delete *cur* from the *Bv* genome | This study |
| 1619 | dBvu_3580_5r | TATGAATTGATATTAGATTAAACGTGCTATAAAACCGTGTTATAGAGC |  | This study |
| 1620 | dBvu_3580_3f | AATCTAATATCAATTCATACTTCACACACACAGG |  | This study |
| 1623 | pSIE1-dBvu_3580_3r | TCCACCGCGGTGGCGGCCGCTCTTCTGCCATAATATGTTTGGCA |  | This study |
| 1624 | pSIE1-dBACOVA_05152_5f | GATTAGCATTATGAGGATCCAACAATTATCGCCTCCGAACC | Constructing a plasmid to delete *cur* from the *Bo* genome | This study |
| 1625 | dBACOVA_05152_5r | ATTCATTTTTACCATGAGTGCTACAAAACTG |  | This study |
| 1626 | dBACOVA_05152_3f | ACTCATGGTAAAAATGAATGAGAGTTTCTGCACCAATCGTTC |  | This study |
| 1627 | pSIE1-dBACOVA_05152_3r | TCCACCGCGGTGGCGGCCGCCACGATAGTTCTTTTCCTCTTCGG |  | This study |
| 2624 | pKO_BACOVA_04371f | GCTCTAGAACTAGTGGATCCGATTTGACGGATTTTCTGAAAAAGTCAC | Constructing a plasmid to C-terminally HA tag *BACOVA_04371* | This study |
| 2625 | pKO_BACOVA_04371-HAr | CCCCCTCGAGGTCGACTTAAGCGTAGTCTGGGACGTCGTATGGGTACTCTTTTTTCCGTTCCTGTTTTCTC |  | This study |
| 2664 | lyl-BT16Sf | GTGAATTCGAGCTCGGTACCCAGTACTGCTTGACCATAAGAAC | Constructing a multi-copy plasmid incorporating the 16s promoter upstream of a MCS | This study |
| 2665 | lyl-BT16Sr | AGGTCGACTCTAGAGGATCCACTGCAAAGATAAGAACTTTTAGC |  | This study |
| 2666 | 16S-BTnc140f | GCTAAAAGTTCTTATCTTTGCAGTATAACAGTCTGCGATATATCTTATCG | Constructing a multicopy plasmid to express *BTnc140* from the 16s promoter | This study |
| 2667 | lyl-BTnc140r | TTGCATGCCTGCAGGTCGACAAAAAGAAAAAAGGACGCAACACTATC |  | This study |
| 2670 | 16S-BTnc195f | GCTAAAAGTTCTTATCTTTGCAGTGTTGAACTTTAAAGCTAACATTATGGG | Constructing a multicopy plasmid to express *BTnc195* from the 16s promoter | This study |
| 2671 | lyl-BTnc195r | TTGCATGCCTGCAGGTCGACACAAAAAAGAATTCACCACGGACTACCC |  | This study |
| 2674 | 16S-BTnc364f | GCTAAAAGTTCTTATCTTTGCAGTTAACCATAAGCGAAAAAGTATAAAATGAAGTC | Constructing a multicopy plasmid to express *BTnc364* from the 16s promoter | This study |
| 2675 | lyl-BTnc364r | TTGCATGCCTGCAGGTCGACAATAGGAATAGCTGGCAGGAAG |  | This study |
| W3595 | dBT2131_5f | GCTCTAGAACTAGTGGATCCTGAGCAAGGAGTATTATTGCTCAAC | Constructing a plasmid to delete *BT2131* from the *Bt* chromosome | This study |
| W3596 | dBT2131_5r | TTTACTTTTGGTTTAAAGGTTGATAATTTT |  | This study |
| W3597 | dBT2131_3f | ACCTTTAAACCAAAAGTAAAGTTAACAATTACTTTTCTTACAGAGAGAGTGTG |  | This study |
| W3598 | dBT2131_3r | AAGATAACATTCGAGTCGACTATCTCTGCTAAAATTGTATTTGAGACAGA |  | This study |
| W3702 | dBT1450_5f | GCTCTAGAACTAGTGGATCCCGTCACCGCGGGCATATTTA | Constructing a plasmid to delete *BT1450-BT1448* from the *Bt* chromosome | This study |
| W3703 | dBT1450_5r | AGGCTGTTTGTATTAATGTTATGTGTAAAA |  | This study |
| W3704 | dBT1448_3f | AACATTAATACAAACAGCCTTTCTCAAAAAAAATAAAGGTGTTTTACGTA |  | This study |
| W3705 | dBT1448_3r | AAGATAACATTCGAGTCGACAACTCCGGCGTGCGCATAGA |  | This study |
| W3829 | BT4299KOf | GCTCTAGAACTAGTGGATCCGCACCAGACCAATTCCTATACAAGA | Constructing a plasmid to disrupt *BT4299*. | This study |
| W3830 | BT4299KOr | GGCCCCCCCTCGAGGTCGACAGTGTCTTTATCTTCCGGAGTGATC |  | This study |

Table S3. Standard Mouse Chow Components (5021, Lab Diet)

| **Component** | **Amount** |
| --- | --- |
| **Nutrients (%)** | |
| Protein | 21.5 |
| Arginine | 1.34 |
| Cystine | 0.38 |
| Glycine | 1.02 |
| Histidine | 0.55 |
| Isoleucine | 0.86 |
| Leucine | 1.55 |
| Lysine | 1.25 |
| Methionine | 0.60 |
| Phenylalanine | 0.89 |
| Tyrosine | 0.59 |
| Threonine | 0.79 |
| Tryptophan | 0.25 |
| Valine | 0.99 |
| Serine | 0.94 |
| Aspartic Acid | 2.07 |
| Glutamic Acid | 3.97 |
| Alanine | 1.24 |
| Proline | 1.28 |
| Taurine | 0.04 |
| Fat (ether extract) | 9.8 |
| Fat (acid hydrolysis) | 11.1 |
| Cholesterol, ppm | 295 |
| Linoleic Acid | 2.58 |
| Linolenic Acid | 0.26 |
| Arachidonic Acid | 0.03 |
| Omega-3 Fatty Acids | 0.59 |
| Total Saturated Fatty Acids | 2.58 |
| Total Monounsaturated Fatty Acids | 2.89 |
| Fiber (Crude) | 3.6 |
| Neutral Detergent Fiber | 14.7 |
| Acid Detergent Fiber | 4.4 |
| Nitrogen-Free Extract (by difference) | 49.5 |
| Starch | 29.0 |
| Sucrose | 1.04 |
| Total Digestible Nutrients | 81.1 |
| Gross Energy, kcal/gm | 4.62 |
| Physiological Fuel Value, kcal/gm | 3.72 |
| Metabolizable Energy, kcal/gm | 3.35 |
| **Minerals (%)** | |
| Ash | 5.5 |
| Calcium | 0.80 |
| Phosphorus | 0.82 |
| Phosphorus (non-phytate) | 0.47 |
| Potassium | 0.84 |
| Magnesium | 0.22 |
| Sulfur | 0.27 |
| Sodium | 0.28 |
| Chloride | 0.46 |
| Fluorine, ppm | 13 |
| Iron, ppm | 200 |
| Zinc, ppm | 130 |
| Manganese, ppm | 140 |
| Copper, ppm | 17 |
| Cobalt, ppm | 0.53 |
| Iodine, ppm | 1.6 |
| Chromium (added), ppm | 0.01 |
| Selenium, ppm | 0.45 |
| **Vitamins (ppm)** | |
| Carotene | 0.3 |
| Vitamin K | 3.2 |
| Thiamin | 86 |
| Riboflavin | 8.0 |
| Niacin | 87 |
| Pantothenic Acid | 22 |
| Chlorine | 1590 |
| Folic Acid | 3.0 |
| Pyridoxine | 15 |
| Biotin | 0.30 |
| B12, mcg/kg | 51 |
| Vitamin A, IU/gm | 30 |
| Vitamin D (added), IU/gm | 3.4 |
| Vitamin E, IU/kg | 59 |
| Ascorbic Acid, mg/gm | 0.0 |
| **Calories provided by: (%)** | |
| Protein | 23.107 |
| Fat (ether extract) | 23.698 |
| Carbohydrates | 53.195 |

Table S4. Modified Carbohydrate (69%) Mouse Chow Components (S4944, Bio-Serv)

| **Component** | **Amount** |
| --- | --- |
| **Ingredient (gram %)** | |
| Casein | 20 |
| L-Cystine | 0.3 |
| Glucose | 34.9743 |
| Sucrose | 34.9743 |
| Cellulose | 5 |
| Choline Bitartrate | 0.25 |
| Mineral Mix (AIN-93G; below) | 3.5 |
| Vitamin Mix (AIN-93; below) | 1.0 |
| tBHQ | 0.0014 |
| **Minerals (gm/kg)** | |
| Calcium | 5.1 |
| Chloride | 1.6 |
| Copper | 6.0 |
| Chromium | 1.0 |
| Fluoride | 1.0 |
| Iodine | 0.21 |
| Iron | 37.2 |
| Magnesium | 0.51 |
| Manganese | 10.5 |
| Phosphorus | 2.8 |
| Potassium | 3.6 |
| Selenium | 0.17 |
| Sodium | 1030 |
| Sulfur | 301 |
| Zinc | 37.7 |
| **Vitamins (mg/kg)** | |
| Ascorbic Acid | 0.0 |
| Biotin | 0.20 |
| Choline | 1028 |
| Folic Acid | 2.0 |
| Niacin | 30.0 |
| Pantothenic Acid | 14.7 |
| Pyridoxine | 5.8 |
| Riboflavin | 6.0 |
| Thiamin | 6.0 |
| Vitamin A (IU/kg) | 4140 |
| Vitamin B12 (mcg/kg) | 25 |
| Vitamin D3 (IU/kg) | 1000 |
| Vitamin E (IU/kg) | 83.5 |
| Vitamin K1 (Phylloquinone) (mg/kg) | 0.88 |

**Supplementary References**

1. Koropatkin NM, Martens EC, Gordon JI, et al. Starch catabolism by a prominent human gut symbiont is directed by the recognition of amylose helices. Structure. 2008 Jul;16(7):1105-15.

2. Townsend GE, 2nd, Han W, Schwalm ND, 3rd, et al. Dietary sugar silences a colonization factor in a mammalian gut symbiont. Proc Natl Acad Sci U S A. 2019 Jan 2;116(1):233-238.

3. Townsend GE, 2nd, Han W, Schwalm ND, 3rd, et al. A Master Regulator of Bacteroides thetaiotaomicron Gut Colonization Controls Carbohydrate Utilization and an Alternative Protein Synthesis Factor. mBio. 2020 Jan 28;11(1).

4. Siegel LS, Hylemon PB, Phibbs PV, Jr. Cyclic adenosine 3',5'-monophosphate levels and activities of adenylate cyclase and cyclic adenosine 3',5'-monophosphate phosphodiesterase in Pseudomonas and Bacteroides. J Bacteriol. 1977 Jan;129(1):87-96.

5. Cho KH, Salyers AA. Biochemical analysis of interactions between outer membrane proteins that contribute to starch utilization by Bacteroides thetaiotaomicron. Journal of Bacteriology. 2001 Dec;183(24):7224-7230.

6. Goodman AL, McNulty NP, Zhao Y, et al. Identifying genetic determinants needed to establish a human gut symbiont in its habitat. Cell Host Microbe. 2009 Sep 17;6(3):279-89.

7. Bencivenga-Barry NA, Lim B, Herrera CM, et al. Genetic Manipulation of Wild Human Gut Bacteroides. J Bacteriol. 2020 Jan 15;202(3).

8. Garcia-Bayona L, Comstock LE. Streamlined Genetic Manipulation of Diverse Bacteroides and Parabacteroides Isolates from the Human Gut Microbiota. mBio. 2019 Aug 13;10(4).

9. Van Tassell RL, Lyerly DM, Wilkins TD. Purification and characterization of an enterotoxin from Bacteroides fragilis. Infect Immun. 1992 Apr;60(4):1343-50.
